# FCN1 (M-ficolin), which directly associates with immunoglobulin G1, is a molecular target of intravenous immunoglobulin therapy for Kawasaki disease

**DOI:** 10.1038/s41598-017-11108-0

**Published:** 2017-09-12

**Authors:** Daisuke Okuzaki, Kaori Ota, Shin-ichi Takatsuki, Yukari Akiyoshi, Kazuyuki Naoi, Norikazu Yabuta, Tsutomu Saji, Hiroshi Nojima

**Affiliations:** 1 DNA-chip Development Center for Infectious Diseases, Research Institute for Microbial Diseases Osaka University, 3-1 Yamadaoka, Suita, Osaka 565-0871 Japan; 2 Department of Molecular Genetics, Research Institute for Microbial Diseases, Osaka University, 3-1 Yamadaoka, Suita, Osaka 565-0871 Japan; 3 Department of Pediatrics, Toho University Omori Medical Center, 6-11-1 Omori-nishi, Ohta, Tokyo 143-8541 Japan; 4 Fukae Kasei Co., Ltd., 2-2-7 Murotani, Nishi-ku, Kobe, Hyogo 651-2241 Japan

## Abstract

Kawasaki disease (KD), an acute systemic vasculitis of early childhood, is of unknown etiology. High-dose intravenous immunoglobulin (IVIG) is an effective treatment, but its molecular target remains elusive. DNA microarray analysis of peripheral blood mononuclear cells (PBMCs) revealed that at least 21 genes are drastically down-regulated after IVIG treatment in most KD patients. qRT-PCR analysis confirmed that the mRNA levels of five of these genes were considerably reduced in almost all KD patients after IVIG treatment. Western blot (Wb) of PBMC extracts revealed that levels of FCN1 (M-ficolin), a protein of the complement system that defends against infectious agents, were reduced after IVIG treatment in many KD patients. In another set of KD patients, Wb confirmed that levels of both FCN1 were greatly reduced after IVIG therapy. Wb revealed that the collagen-like domain of FCN1 directly bound to IgG1 *in vitro* through a portion of the CH1 and CH3 domains, and synthetic peptides corresponding to these domains of IgG1 efficiently inhibited these associations. These results suggest that FCN1 is a molecular target of intravenous IVIG in KD patients. We propose that these peptides and a humanized monoclonal antibody against FCN1 could be useful in combination therapy with IVIG.

## Introduction

Kawasaki disease (KD) is an acute systemic vasculitis of unknown etiology that occurs primarily in children younger than 5 years of age. KD patients suffer from the systemic inflammation of the medium-sized blood vessels^1, 2^. Because it is frequently associated with the development of coronary artery abnormalities such as vasculitis of arteries, veins, and capillaries, KD is the leading cause of acquired heart disease in childhood^3^. To date, the causative agents of KD have not been identified^4, 5^. The frontline therapy for KD is high-dose intravenous immunoglobulin (IVIG); early IVIG therapy in the acute phase effectively reduces the incidence of coronary artery abnormality, thereby preventing the most serious cardiac defects^6^.

IVIGs are manufactured from pooled human plasma from more than one thousand donors per batch, typically containing more than 95% unmodified immunoglobulins (IgGs) with a broad spectrum of specificities and intact Fc (fragment, crystallizable)-dependent effector functions^7^. As the role of IVIG in KD, Fc-specific natural regulatory T cells and immature myeloid dendritic cells, as well as blockade of activating Fc-gamma receptors (FcγR) and stimulation of the inhibitory FcγRIIb receptor, have been proposed; they may be important in the response to IVIG^8, 9^. However, the detailed mechanisms underlying immune regulation by IVIG remain unknown. IVIG therapy has been successfully applied to other autoimmune and systemic inflammatory diseases such as immune thrombocytopenia, Guillain-Barré syndrome, preterm and neonatal sepsis, intractable childhood epilepsy, experimental autoimmune myositis, multifocal motor neuropathy, polymyositis and dermatomyositis, systemic lupus erythematosus, Still’s disease, and antiphospholipid antibody syndrome^6, 10, 11^.

FCN1 (ficolin-1 or M-ficolin) is a member of the complement system, which plays a major role in innate immune defense against infectious agents^12, 13^. We previously reported that the *FCN1* mRNA level is elevated in peripheral blood mononuclear cells (PBMCs) of patients with vasculitis, including Takayasu arteritis (TA) and microscopic polyangiitis (MPA); specifically, we observed elevated expression of FCN1 in macrophages in the inflamed regions of surgical aorta specimens from TA patients^14^ and surgical glomeruli specimens taken from MPA patients^15^. FCN1 is also up-regulated in PBMCs from DBA/2 mice suffering from severe vasculitis following injection with *Candida albicans* water-soluble fraction (CAWS), a putative model mouse of KD^16^. No previous study has investigated whether FCN1 is up-regulated in PBMCs of KD patients.

In this study, we sought to identify the target proteins of IVIG by examining gene-expression profiles in PBMCs of KD patients. To this end, we performed DNA microarray analysis to identify genes whose mRNA levels were up- or down-regulated in PBMCs of most or all KD patients, reasoning that such genes may be linked to the pathogenesis of KD. We successfully identified several genes that were down-regulated after IVIG in almost all KD patients examined. Wb analysis revealed that serum FCN1 levels were drastically reduced after IVIG treatment in 100% of examined KD patients. Wb also revealed that the collagen-like domain of FCN1 directly bound to IgG1 *in vitro* through a portion of the CH1 and CH3 domains, and synthetic peptides corresponding to these domains of IgG1 efficiently inhibited these associations. Based on these findings, we conclude that FCN1 is a molecular target of intravenous IVIG in KD patients.

## Results

### DNA microarray analysis of PBMCs from KD patients

To determine whether the gene-expression profiles of KD patients share common abnormalities relative to those of healthy volunteers (HVs), we subjected RNA samples from PBMCs to genome-wide cDNA microarray analyses using Agilent Hu44K arrays. We performed DNA microarray analyses on RNA samples from PBMCs of 19 individual KD patients (Fig. S1) at the indicated times relative to treatment: day 1 (d1) refers to blood collected before IVIG treatment; d2 or d7 refers to blood collected at 2–3 days or 6–8 days after treatment, respectively (convalescent stage). At 6–8 days after IVIG treatment, symptoms of all responder patients, but not non-responder patients, were improved.

**Figure 1:**
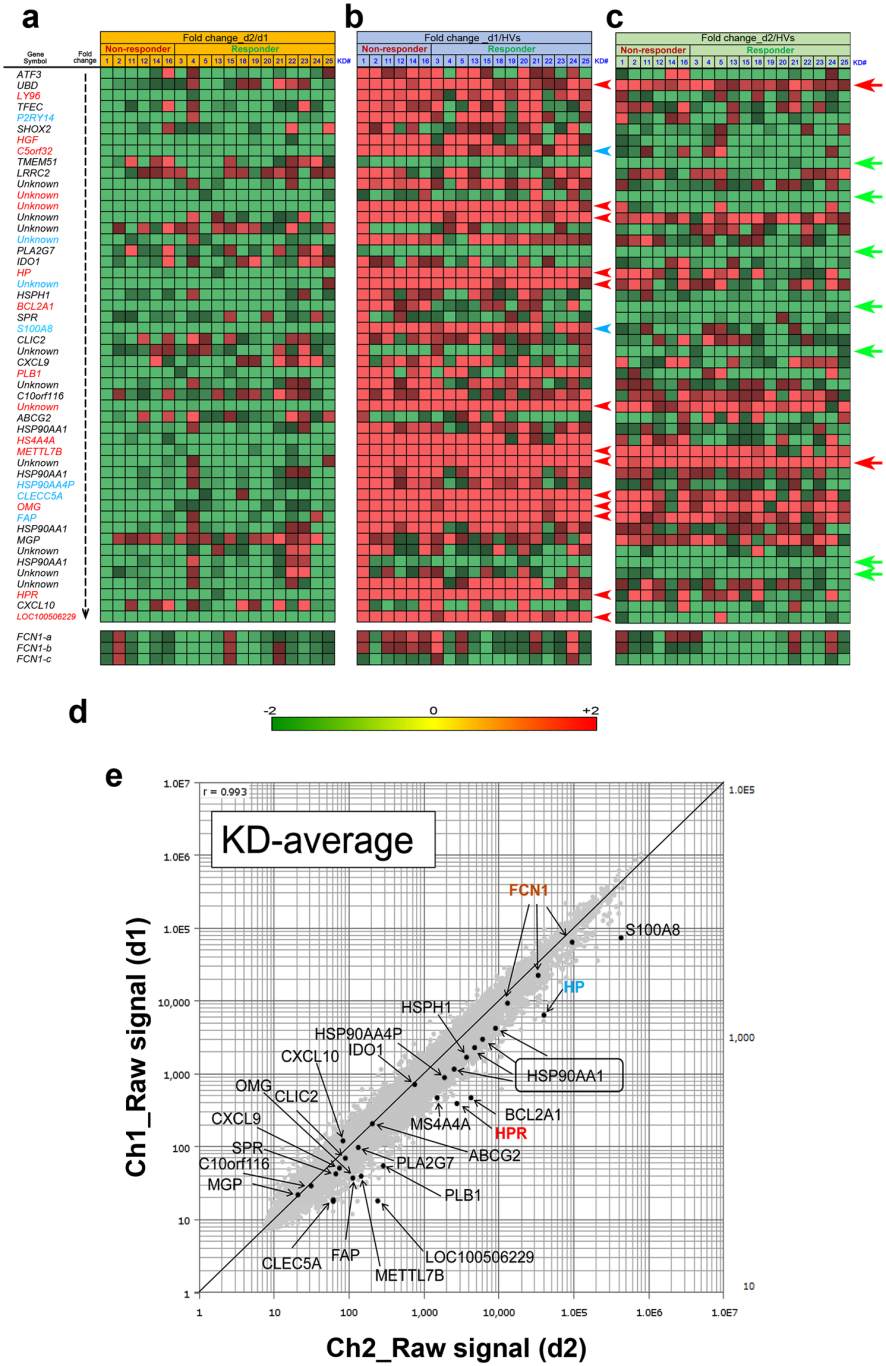
Expression profiles of genes whose mRNA levels were commonly up- or down-regulated in PBMCs of 19 KD patients. (**a)** List of the 50 genes arranged in the descending order of averaged fold change values of signal intensity (d2 vs d1); mosaic tile representation of each gene is also shown. (**b**) Mosaic tile representation for each listed gene when the fold change of signal intensity was compared (d1 vs HVs). (**c**) Mosaic tile representation for each listed gene when the fold change of signal intensity was compared (d2 vs HVs). Colors are defined in **(d)** “Unknown” indicates uncharacterized genes. Names of notable genes are highlighted in blue and red font with by green and red arrows. Tiles corresponding to three independent probes for FCN1 are shown at the bottom. **(d)** Color values in (**a**–**c**) indicating mean expression levels (expressed as log_2_[fold change]): green, down-regulation; red, up-regulation. **(e)** Scatter plots for average values from 19 KD patients. The y-axis and x-axis show log_2_[hybridization signal intensity] of microarray data from d1 and d2, respectively.

We first compared signal intensities before and after IVIG treatment (i.e., the fold change at d2 vs d1) (Fig. 1a–d). Because the microarray possessed three independent probes for *FCN1* with slightly distinct signal intensities, we displayed these data separately at the bottom of Fig. 1a–c. At least 21 genes were drastically down-regulated (green tiles) by IVIG treatment in almost all KD patients (Fig. 1a): 14 genes (red font) were down-regulated in every patient, and seven genes (blue font) were down-regulated in all but one case each. Comparison of signal intensities between KD patients at d1 and the average intensities from HVs (Fig. 1b) revealed that 12 genes were up-regulated in all KD patients (red arrowhead), and four genes (blue arrowhead) were down-regulated in all but one case each. Comparison between d2 and HVs revealed that two genes were up-regulated (red arrows) and seven were down-regulated (green arrows) in all KD patients (Fig. 1c).

To determine whether these changes in mRNA levels were physiologically significant, we generated a scatter plot (d1 vs d2) in order to analyze their distribution at a glance (Fig. 1e, Fig. S2). Because fold changes of compared genes with raw signal intensity >100 at least in one of the samples are generally considered to be physiologically significant, we focused our subsequent analysis on haptoglobin (*HP*), haptoglobin-related (*HPR*), *FCN1* ^12, 13^, fibroblast activation protein (*FAP*), oligodendrocyte-myelin glycoprotein (*OMG*), methyltransferase-like 7B (*METTL7B*), membrane-spanning 4-domains, subfamily A, member 4 A (*MS4A4A*), S100 calcium binding protein A8 (*S100A8*), and a putative non-coding RNA (*LOC100506229*). Genes of unknown function were omitted from this analysis.

### Quantitative reverse transcription-polymerase chain reaction (qRT-PCR)

**Figure 2:**
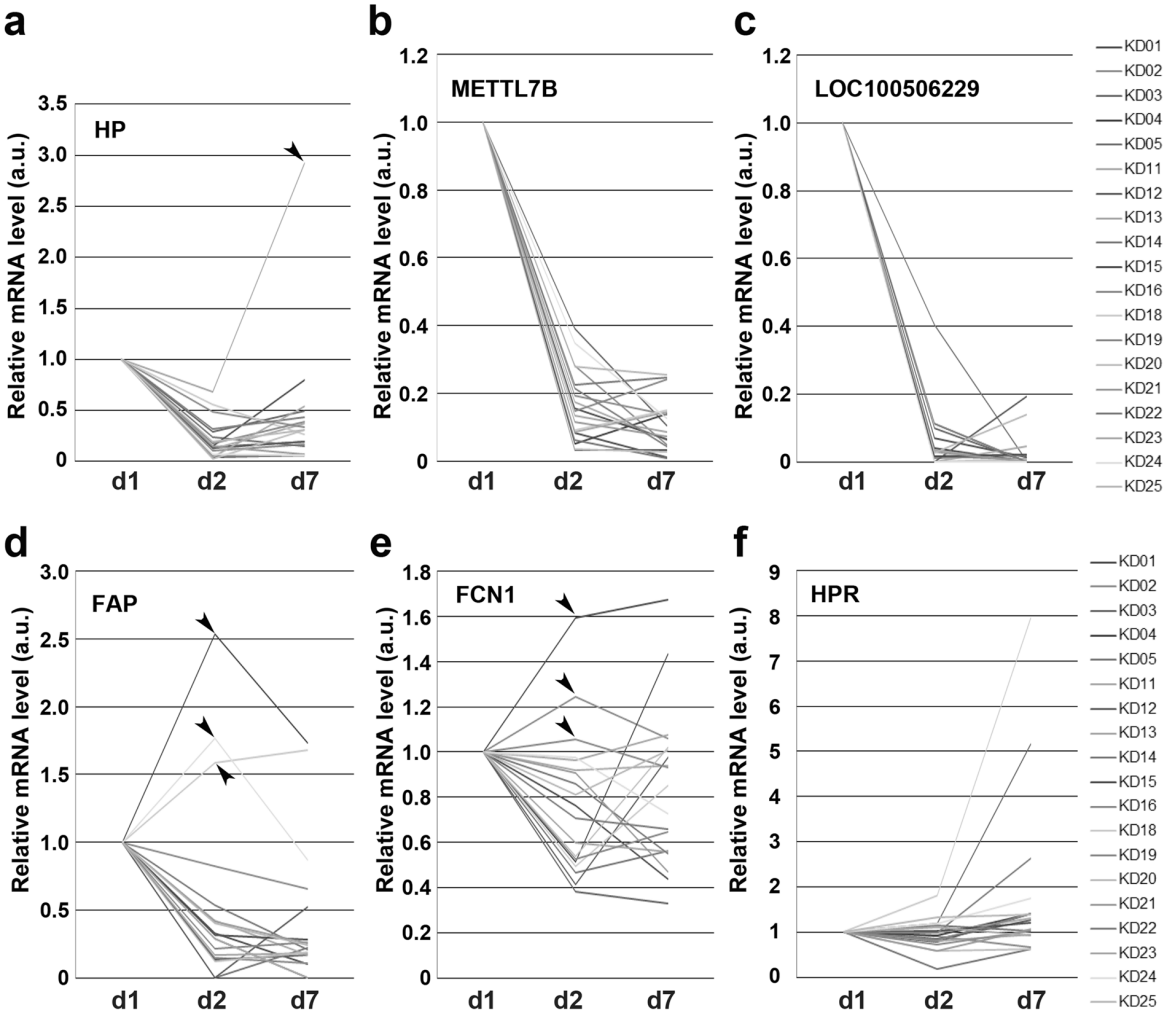
Line graph of qRT-PCR data. Relative mRNA levels of *HP* (**a**), *METTL7B* (**b**), *LOC100506229* (**c**), *FAP* (**d**), *FCN1* (**e**) and *HPR* (**f**) were determined by qRT-PCR using purified RNA from each KD patient (indicated in the rightmost column) at 1 day before IVIG treatment (d1), 2–3 days after IVIG treatment (d2), and 6–8 days (d7) after IVIG treatment. The vertical axis indicates mRNA level (arbitrary units, a.u.) relative to that at 1d, which was fixed at 1.0 a.u. Arrowheads denote exceptional cases.

**Figure 3:**
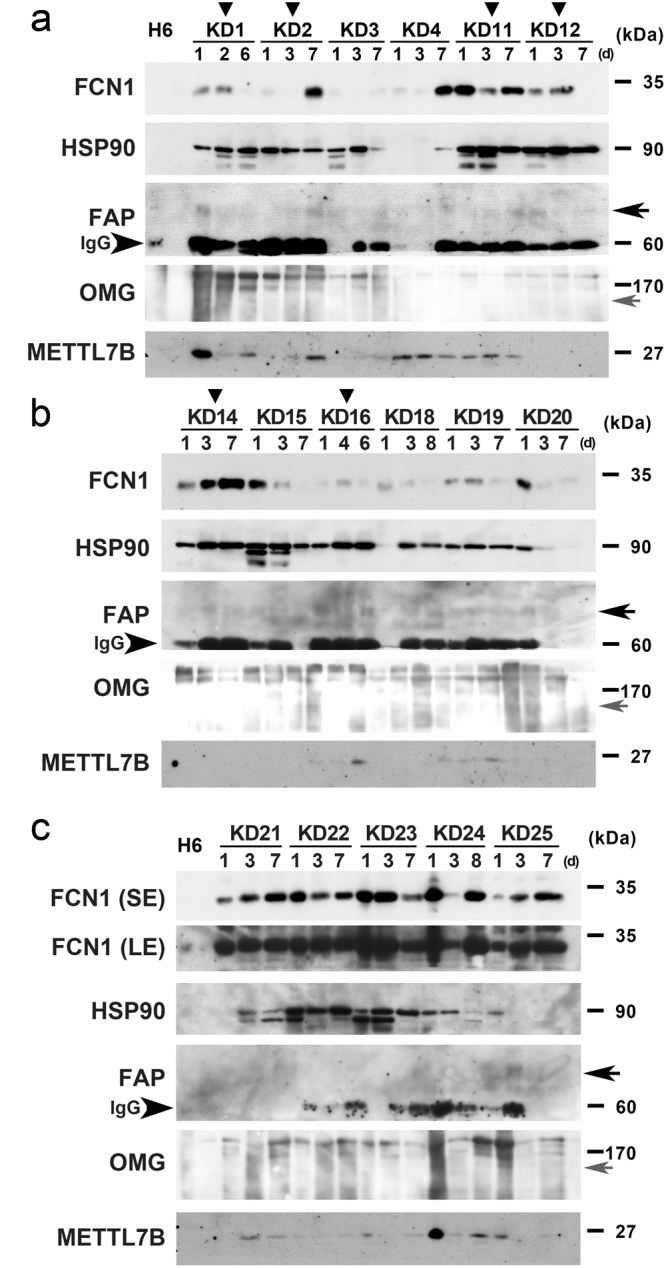
Wb analysis to detect expression levels of the indicated proteins in PBMCs of KD patients. (**a–c**) Protein levels of PBMCs from healthy volunteers (H6) were used to illustrate altered expression levels in KD patients. Number for each lane (d; days) indicates the date when blood was collected from each patient. Large and small arrows indicate the putative bands for FAP and OMG, respectively. For FCN1, images obtained by short exposure (SE) or long exposure (LE) are displayed (**c**). Vertical arrowheads denote non-responders to IVIG therapy. Horizontal arrowhead indicates the band for immunoglobulin (IgG) that served as a loading control.

To confirm that the microarray signals accurately reflected mRNA levels in PBMCs of KD patients, we performed qRT-PCR on the *HP*, *METTL7B*, *LOC10050622*9, *FAP*, *FCN1*, and *HPR* genes using the same RNA samples used for the microarray analysis. Line graph (Fig. 2), dot plot (Fig. S3) and bar graph (Fig. S4) presentations revealed that *HP*, *METTL7B*, and *LOC100506229* mRNA levels were drastically lower at d2 than at d1 in all KD patients; these changes are statistically significant (Fig. S3). With the exception of *HP* in one patient (arrowhead in Fig. 2a), these low mRNA levels were maintained at d7. These results validated the accuracy of our DNA microarray analysis (see Fig. 1a–c). In the case of *FAP*, all but three patients (arrowheads in Fig. 2d) had reduced mRNA levels at d2, whereas the *FCN1* mRNA level exhibited a modest decrease at d2 in all but three (different) patients (arrowheads in Fig. 3e). By contrast, the *HPR* mRNA level was not reduced at d2 in most patients, i.e., the microarray data were not reproducible for this gene (Fig. 3f).

The dot plots in Fig. S3, where the data were normalized against the average value of 19 KD patients (horizontal bar in each dot plot), highlight the differences in overall distributions of relative mRNA levels between d1, d2, and d7 (Fig. S3). The *HP*, *METTL7B*, and *LOC10050622*9 mRNA levels were conspicuously lower at d2 than at d1; all of these differences are statistically significant (Fig. S3a–c). The *HPR* mRNA level was almost unchanged between 1d, d2, and d7 (Fig. S3d), whereas average *FAP* and *FCN1* mRNA levels were reduced at d2 (Fig. S3e,f).

The bar graphs of raw data in Fig. S4 emphasize the elevation of *HP*, *METTL7B* and *LOC100506229* mRNA levels in each KD patient relative those in HVs, shown in the leftmost columns (Fig. S4a–c). They also accentuate the reduction mRNA levels in each patient at d2 relative to 1d. By contrast, *FAP* and *FCN1* mRNA levels were only 1–5-fold higher in patients at d1 than in HVs (Fig. S4e,f), and *HPR* levels were lower in patients than in HVs (Fig. S4f). According to these results, the qRT-PCR analysis validated the accuracy of the microarray data for *HP*, *METTL7B*, *LOC100506229*, *FAP* and *FCN1*; however, the microarray data for *HPR* was not reproducible.

### Wb analysis for proteins in PBMCs

To determine whether alterations in mRNA levels affected the corresponding protein levels, we performed Wbs on PBMC extracts from each KD patient. For this purpose, we selected proteins that could be detected using commercially available antibodies: Hp, HpR, FCN1, HSP90, FAP, OMG, METTL7B, and S100A8. Hp and HpR levels in PBMCs varied among patients, but were higher in all KD patients at d1 than in HVs (HV1 or HV4) (Fig. S5a). Comparison of protein levels between d1, d2, and d7 in each KD patient revealed that Hp levels in 6 of 17 KD patients (KD1, -4, -12, -20, -24 and -25) were dramatically lower at d2 than at d1 (Fig. S5a, top panel), indicating that Hp protein and mRNA levels were not perfectly correlated in these KD patients. Notably, 9 (KD2, -3, -4, -11, -12, -15, -18, -20, -22, and -24) and 7 (KD1, -3, -12, -15, -16, -20, and -24) of 17 KD patients also exhibited a conspicuous decrease in the levels of FCN1 and FAP (large arrows in Fig. 3), respectively, at d2 relative to d1, suggesting that PBMC protein levels of FCN1 and FAP were affected by IVIG therapy in many KD patients. By contrast, only four patients (KD1, -12, -24 and -25) had reduced HpR levels at d2 (Fig. S5a, lower panel), and only four patients (KD1, -23, -24 and -25) and two patients (KD1 and -23) had reduced levels of METTL7B and HSP90, respectively (Fig. 3). OMG protein was only faintly detectable in PBMCs (small arrows in Fig. 3). These results indicate that levels of FCN (and, less markedly, Hp and FAP) were reduced following IVIG therapy in PBMCs of many KD patients.

### FCN1 in serum of KD patients were almost depleted after IVIG

**Figure 4:**
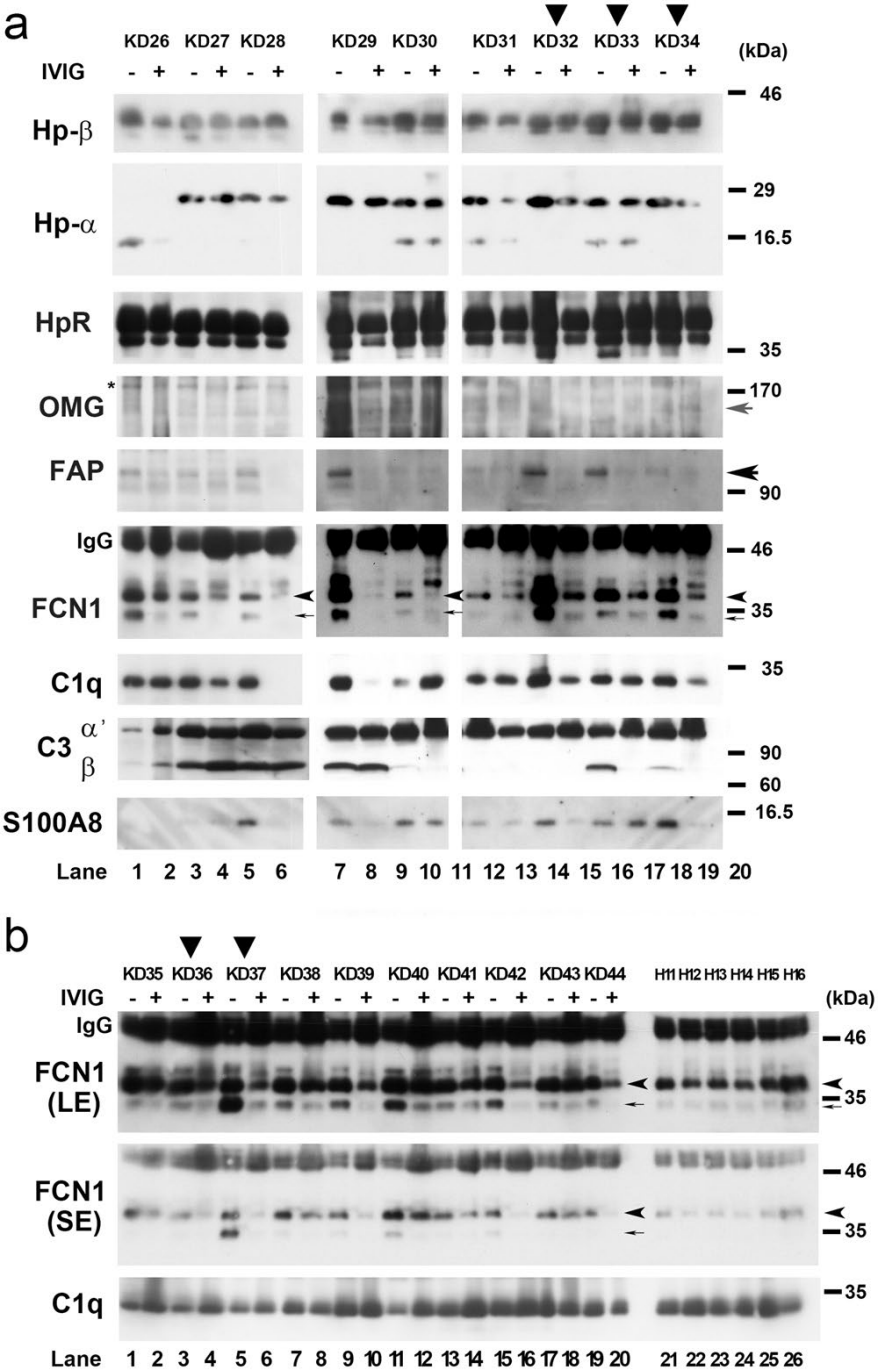
Wb analysis to detect the indicated proteins in serum of KD patients. (**a**,**b**) Vertical arrowheads denote the non-responders to IVIG therapy. Thin arrow and small arrowhead denote the bands for FCN1 and FCN1 precursor, respectively. Small gray arrow, large black arrow, and arrowhead indicate the bands for OMG, FAP, and FCN1, respectively. Asterisks denote putative background bands. IgG indicates the band for immunoglobulin (IgG), which was used as a loading control. Serum was collected from KD patients before (−) or 2–3 days after (+) IVIG treatment. For FCN1 Wbs from additional KD patients (**b**), images obtained by short exposure (SE) or long exposure (LE) are displayed. H11–H16 denote new healthy volunteers. We used an equal amount of serum for Wb analysis on each patient, which was considered as a loading control.

Knowledge of the influence of IVIG therapy on the serum protein level is important for understanding its therapeutic impact on KD patients. To determine whether the levels of the proteins described above were also altered in serum of KD patients following IVIG therapy, we collected serum from an additional set of KD patients (KD26-KD34) before (−) and after ( + ) IVIG treatment, and then subjected these samples to Wb (Fig. 4a); the total number of enrolled patients is thirty-eight (Fig. S1a) because we enrolled 19 patients (KD1~KD25) for DNA microarray (Fig. 1) and Wb (Fig. 2) analyses PBMC and 19 patients (KD26~KD44) for this analysis. Notably, the band intensities of FCN1 (thin arrow) and FCN1 precursor (arrowhead) were significantly reduced after IVIG therapy in all nine of these KD patients; the FCN1 precursor band was not detectable in PBMC samples (see Fig. 4). The FAP level was also reduced after IVIG therapy in seven of the nine KD patients (large arrow). By contrast, Hp-α and Hp-β levels were reduced only in five patients (KD26, KD31, KD32 and KD34), and the S100A8 level was reduced only in four patients (KD28, KD29, KD32 and KD34). The HpR level was almost unaltered in all patients, and the OMG level was too low to judge alterations. Because IVIG reduces serum concentrations of complement factor C1q and C3 in multifocal motor neuropathy patients^17^, we monitored the concentrations of these proteins by Wb. However, serum C1q or C3 level was reduced in only five (KD27, KD28, KD29, KD31 and KD33) or three (KD28, KD31 and KD33) of nine KD patients, respectively.

To determine whether the FCN1 level was also reduced in other KD patients, we examined ten additional patients, all of whom (19/19 = 100%) exhibited lower levels of both FCN1 (thin arrow) and FCN1 precursor (arrowhead) after (+) IVIG treatment (lanes 1–20 in Fig. 4b). By contrast, both FCN1 and FCN1 precursor levels were lower in HVs (H11–H16) than in KD patients prior to IVIG therapy, suggesting that IVIG normalizes the serum FCN1 level in KD patients (Fig. 4b). Previous work showed that the C1q level is not reduced in KD patients^14^; consistent with this observation, in our samples the C1q level was similar among KD patients and HVs; therefore, we conclude that C1q is not involved in the response to IVIG. On the other hand, the remarkable reduction in the serum FCN1 level in KD patients after IVIG treatment suggests that FCN1 is an important molecular target of IVIG therapy in KD patients.

### The collagen-like domain of FCN1 binds to IgG1 *in vitro*

**Figure 5:**
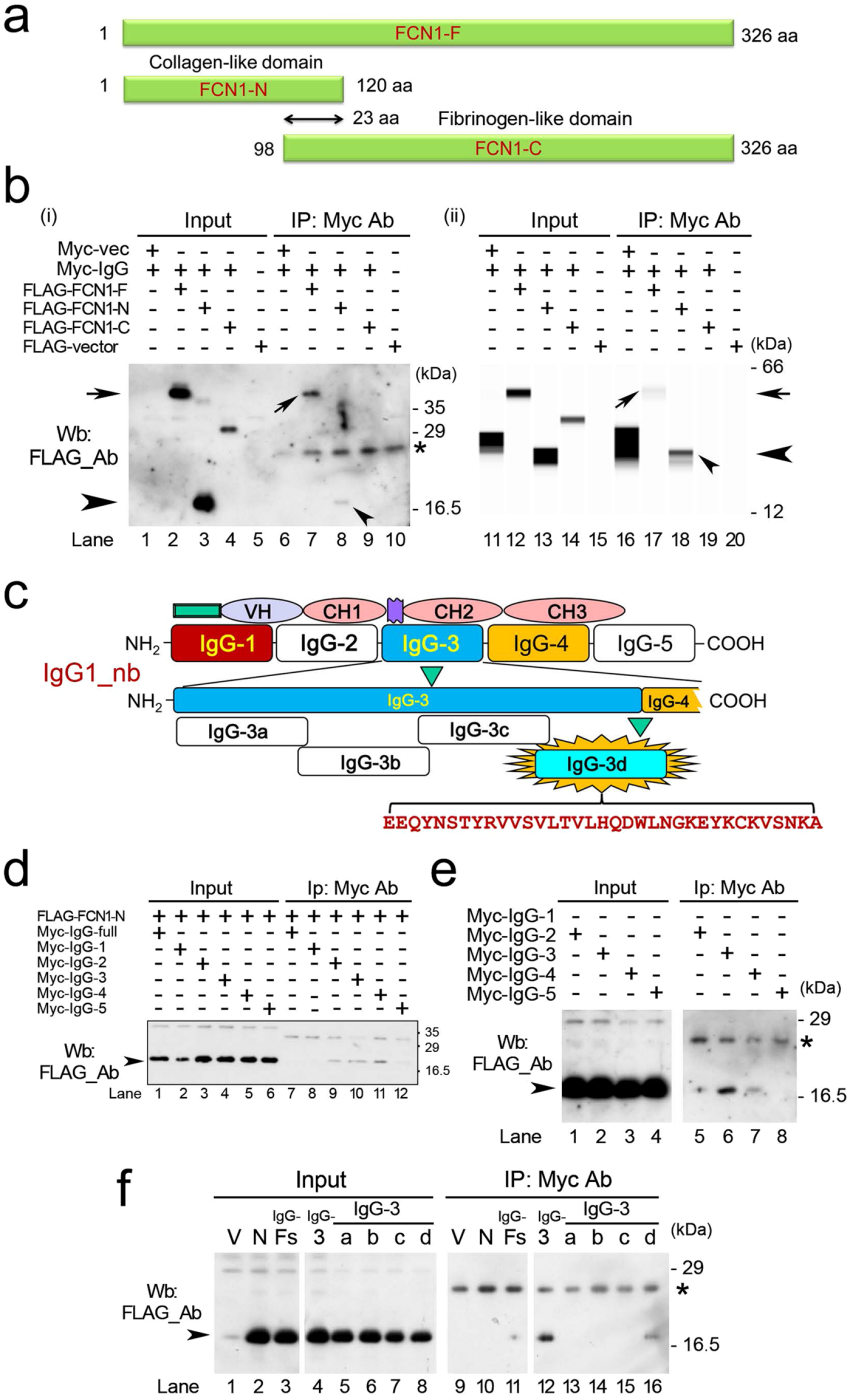
The collagen-like domain of FCN1 associates with IgG. (**a**) Schematic representation of FCN1-F (full size), FCN1-N (collagen-like domain), and FCN1-C (fibrinogen-like domain). (**b**) Wb was performed on extracts from 293 T cells in the presence (+) or absence (−) of the indicated plasmid DNA. Input (lanes 1–5 and 11–15) refers to the extract used for immunoprecipitation (IP) by anti-Myc antibody (Ab; lanes 6–10 and 16–20). Arrows and arrowheads indicate the bands for FCN1-F and FCN1-N, respectively. (**c**) Schematic representation of human IgG1 protein and its cDNA (NEB). The IgG-3 domain was further dissected into four domains (IgG-3a, IgG-3b, IgG-3c, and IgG-3d). (**d**–**f**) Wb was performed on extracts from 293 T cells in the presence (+) or absence (−) of the indicated plasmid DNA. ‘Input’ refers to the extract subjected to IP with anti-Myc antibody. Arrowheads indicate the bands for FCN1-N. Asterisks indicate the putative IgG1 band derived from the IP. V, vector. N, FCN1-N. IgG-Fs, IgG1 full size.

The results described above indicate that FCN1 protein directly interacts with IgG1. To investigate this possibility, we prepared plasmids encoding FLAG-tagged full-size FCN1 (FCN1-F), the N-terminus only (FCN1-N), or the C-terminus only (FCN1-C) (Fig. 5a, S6). We also obtained a plasmid (IgG1-nb; Fig. S7) that expresses Myc-tagged IgG1 protein with one variable region (VH1) and three constant regions (CH1–3). We transfected these constructs into 293 T cells, prepared cell extracts, and immunoprecipitated Myc-IgG1 using anti-Myc antibody. Wb of the immunoprecipitates (IP/Wb) with anti-FLAG antibody detected FCN1-F (arrow) and FCN1-N (arrowhead) proteins, suggesting that both FCN1-F and FCN1-N associated with Myc-IgG1 (Fig. 5b-i). An automated blot analysis system confirmed the direct association between FCN1-N and Myc-IgG1 (Fig. 5b-ii). To narrow down the region of IgG1 that associates with FCN1-N, we prepared plasmids encoding Myc-tagged dissected IgG1 proteins (IgG-1, IgG-2, IgG-3, IgG-4, and IgG-5; Fig. S8) and introduced these constructs into 293 T cells (Fig. 5c-i). IP/Wb revealed that FCN1-N associated with both IgG-2, IgG-3, and IgG-4 fragments at similar levels (lanes 9, 10, and 11 in Fig. 5d). To avoid saturation of the binding due to use of excess protein, we used 4-fold less extract in the IP. In this experiment, Wb detected the strongest band for IgG-3, suggesting that it had the highest affinity for FCN1-N (lane 6 in Fig. 5e). We further dissected the IgG-3 fragment into four fragments (Myc-IgG-3-a, -b, -c, and -d; Fig. S9) and performed similar IP/Wb analysis. Myc-IgG-3-d bound most intensely to FCN1-N (lane 16 in Fig. 5f). These results suggest that FCN1-N associates with Myc-IgG through the Myc-IgG-3-d domain.

### Identification of the domain of IgG1 that associates with FCN1

**Figure 6:**
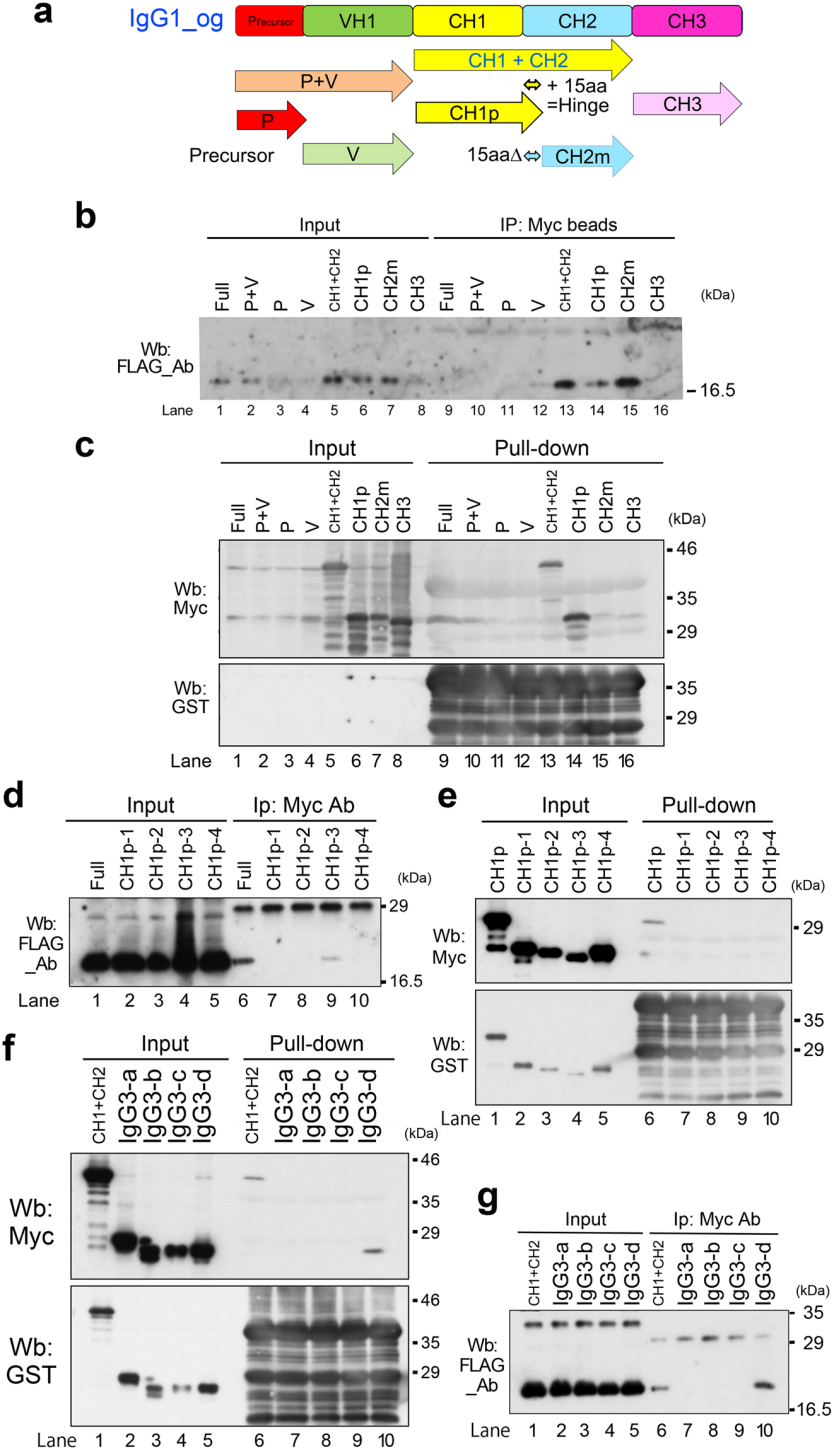
Determination of the association domain between the collagen-like domain of FCN1 and IgG1. (**a**) Schematic representation of human IgG1 protein and its cDNA (OriGene). CH1p and CH1m peptide contain the CH1 domain plus (p) or minus (m) 15 amino acids (15aa) corresponding to the J chain. (**b**,**d**,**g**) Wb was performed on extracts from 293 T cells harboring the indicated plasmid DNA. ‘Input’ refers to the extract subjected to IP with anti-Myc antibody. Arrowheads indicate the bands for FCN1-N. (**c**,**e**,**f**) Wb with anti-Myc antibody and anti-GST antibody (used as a loading control) was performed on immunoprecipitates pulled down with affinity-purified GST-FCN1-N from extracts of 293 T cells harboring the indicated plasmid DNA. ‘Input’ refers to the extract subjected to IP with anti-Myc antibody. Arrowheads indicate bands for FCN1-N. (**d**,**e**) CH1p was dissected into four domains (CH1p1-, CH1p-2, CH1p-3, and CH1p-4). (**f**) Pull-downs of IgG3-a, -b, -c, and -d fragments were also performed to confirm the association of GST-FCN1-N with IgG3-d (lane 10).

To further confirm the FCN1–IgG1 association described above, we prepared a new set of plasmids (IgG1-og; Fig. S10) that encode not only variously dissected fragments of the constant region with the same amino acid (aa) sequences (CH1–3), but also the IgG1 variable region (VH1) with distinct aa sequences from IgG1-nb (Fig. 6a; Fig. S11). IP/Wb indicated that CH1 and CH2 associated with FCN1-N (lanes 13–15 in Fig. 6b). We confirmed this result by performing pull-down experiments using affinity-purified GST-tagged FCN1-N protein (lanes 13 and 14 in Fig. 6c). We then dissected CH1p (CH1 + 15 aa) into four fragments (CH1p-1, -2, -3, and -4; Fig. S12), prepared Myc-tagged plasmid DNA, and performed IP/Wb. We found that CH1p-3 most strongly associated with FCN1-N (lane 9 in Fig. 6d). However, a pull-down experiment failed to detect this association (lanes 2 and 7–10 in Fig. 6e), probably due to steric hindrance. However, we detected the association of GST-FCN1-N with Myc-IgG-3-d in both pull-down experiments (lane 10 in Fig. 6f) and IP/Wb (lane 10 in Fig. 6g). These differences from the aforementioned results (see Fig. 5) may also be due to steric hindrance due to the distinct three-dimensional structures of the IgG1 fragments. Based on these findings, we conclude that FCN1-N associates with IgG1 through the IgG-3-d and CH1p-3 domains.

### Inhibition of the association between IgG1 and FCN1 by IgG1-derived peptides

**Figure 7:**
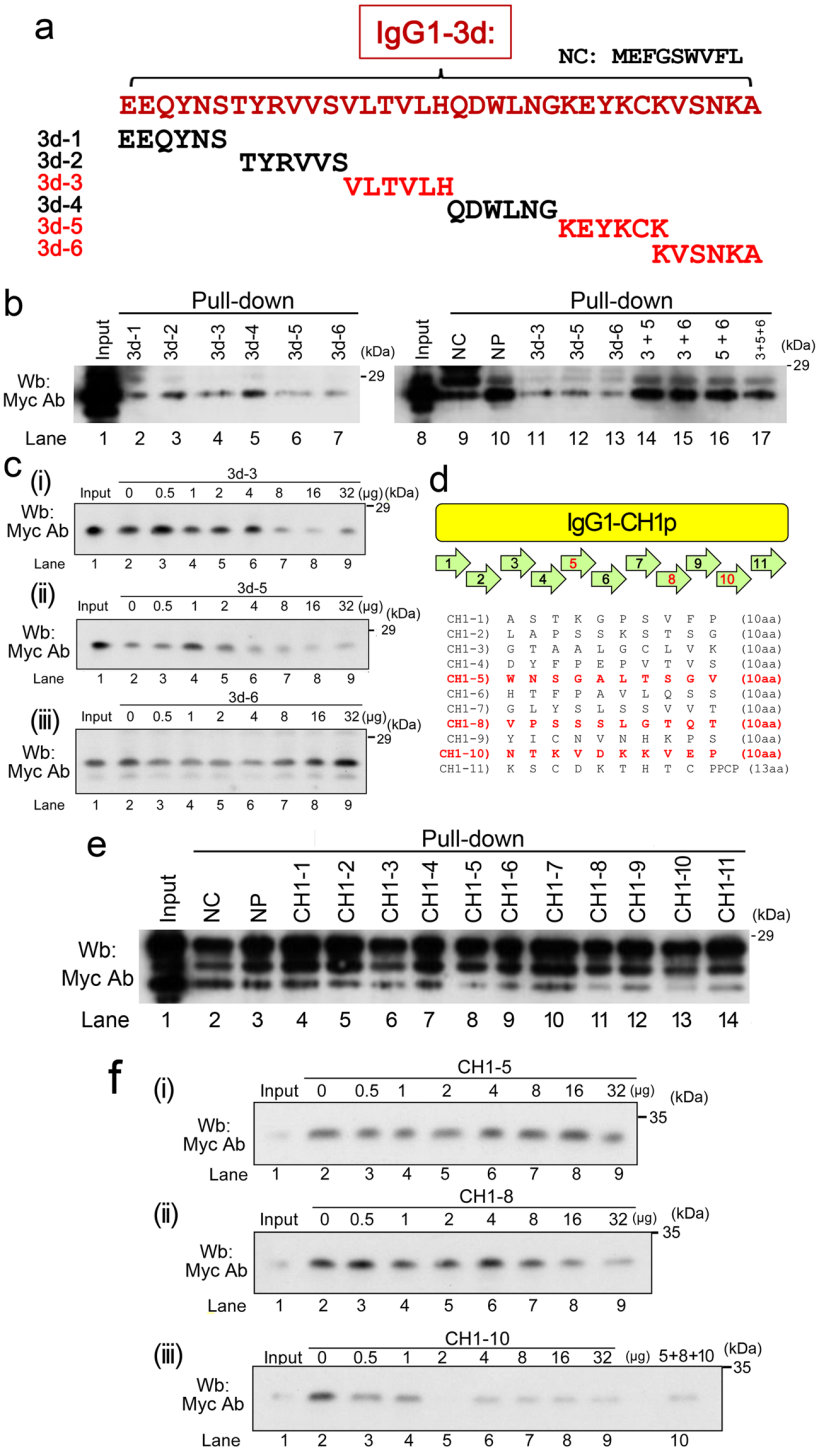
IgG1-derived peptides inhibit the association between FCN1 and IgG1. (**a**) Schematic representation of the location of the peptides derived from the IgG1-3 domain. Red font denotes that these peptides inhibit the association between FCN1 and IgG1. (**b**) Wb was performed to examine the inhibitory effects of the indicated peptides, which were mixed with precipitates of GST-FCN1-N pull-down extracts from 293 T cells expressing IgG3-d. (**c**) Sequential dilutions of potentially inhibitory peptides were mixed with pull-down precipitates to determine the optimal inhibitory concentration. (**d**) Schematic representation of the location of the peptides derived from the IgG1-CH1p domain. Red font indicates inhibitory peptides. (**e**) Wb was performed to examine the inhibitory effects of the indicated peptides, which were mixed with precipitates of GST-FCN1-N pull-down extracts from 293 T cells expressing IgG1-CH1p. (**f**) Sequential dilutions of potentially inhibitory peptides were mixed with pull-down precipitates to determine the optimal inhibitory concentration.

To further confirm the FCN1–IgG1 association, we assessed the inhibitory potency of chemically synthesized peptides. To this end, we first tested six peptides (3d-1, -2, -3, -4, -5, and -6) that cover the IgG-3-d domain (Fig. 7a), and found that 3d-3, −5, and −6 efficiently inhibited the association between GST-FCN1-N and Myc-IgG-3-d (lanes 4, 6, and 7 in Fig. 7b). Although a duplicate experiment confirmed this result (lanes 11–13), any combination of mixed peptides failed to inhibit the association (lanes 14–17), probably due to unexpected interactions between the peptides. Sequential dilution of these peptides indicated that the minimum amounts of 3d-3 (Figs. 7c-i) or 3d-5 (Fig. 7c-ii) required for this inhibition were 8 and 4 μg, respectively, and higher amounts (16 or 32 μg) did not significantly increase the inhibitory effects. Hereafter, we refer to these potentially useful peptides as IVIG-emulating peptide (iViep); iViep1 and iViep2 refer to VLTVLH and KEYCK, respectively. By contrast, inhibition was hindered by larger amounts of 3d-6 peptide (8, 16, or 32 μg), suggesting that this peptide is not effective in this regard (Fig. 7c-iii).

We also performed pull-down experiments to assess the ability of 11 peptides to inhibit the IgG1–CH1p interaction (Fig. 7d). CH1-5, -8, and 10 potently inhibited the association between GST- FCN1-N and IgG1-CH1p (Fig. 7e). Sequential dilution of these peptides revealed that inhibition by CH1-5 was too weak for this peptide to be effective (Fig. 7f-i). The minimum amounts CH1-8 (Fig. 7f-ii) or CH1-10 (Fig. 7f-iii) required for inhibition were 32 and 0.5 μg, respectively. Thus, CH1-10 peptide, hereafter called iViep3, was the most effective. Taken together, these findings demonstrate that association of FCN1-N with IgG1 is very efficiently competitively inhibited by the iViep1, iViep2, and iViep3 peptides.

## Discussion

In this study, we used DNA microarray analysis to show that more than 21 genes were drastically down-regulated after IVIG treatment (d1 versus d2) in PBMCs of almost all KD patients examined (Fig. 1a–d). A scatter plot analysis suggested that the changes in *HP*, *HPR*, *FAP*, *OMG*, *METTL7B*, *MS4A4A*, *S100A8*, *FCN1*, and *LOC100506229* mRNA levels were physiologically significant (Fig. 1e, S2). qRT-PCR analysis confirmed that mRNA levels of *HP*, *METTL7B*, *LOC100506229*, *FAP* and *FCN1*, but not *HPR*, were considerably reduced in almost all KD patients after IVIG treatment (Fig. 2, S3, S4). Wb of PBMCs revealed that Hp, HpR, OMG, and METTL7B levels were reduced in only a subset of patients after IVIG treatment (Fig. 3, S5), whereas FCN1 levels were reduced in many of the patients (Fig. 3). Wb of serum confirmed that levels of both FCN1 and FCN1 precursor were greatly reduced after IVIG therapy in 100% of KD patients examined (Fig. 4). The FAP level was also reduced in most KD patients following treatment (Fig. 4). Based on these results, we propose that the primary role of IVIG in KD is to reduce the FCN1 level in the serum of the patients. Since FCN1, as a recognition molecule of the lectin complement pathway, is involved in innate immune defense against infectious agents^12, 13^, our results also suggest a contribution of infectious agents for the pathogenesis of KD.

Given that *FCN1* mRNA is up-regulated in PBMCs of vasculitis patients (TA and MPA), and immunostaining revealed augmented expression of FCN1 in macrophages in the inflamed regions of surgical specimens of TA and MPA patients^14, 15^, augmented FCN1 levels in both PBMCs (Fig. 3) and serum of KD patients (Fig. 4) suggest that FCN1 is somehow related to the pathogenesis of vasculitis in KD. Although it is difficult to obtain surgical aorta specimens from KD patients, immunostaining of such specimens would provide confirmation that the elevated FCN1 level can be at least partially attributed to macrophages. Moreover, Wb of serum obtained before and after IVIG therapy from patients with other autoimmune and systemic inflammatory diseases to which IVIG therapy has been successfully applied could provide support for the generally applicability of our FCN1 findings to other autoimmune diseases. Furthermore, measurement of serum FCN1 levels might provide a useful tool for predicting the efficacy of IVIG therapy in untested systemic inflammatory diseases. We intend to address these topics in future research. Because the physiological role of FAP in vasculitis (including KD) is unknown, a detailed analysis of this protein’s functions should also be performed.

Interestingly, FCN1-N directly bound to IgG1 *in vitro* via the IgG1-3d (Fig. 5) and IgG1–CHp-3 domains (Fig. 6). Three peptides (iViep1, iViep2, and iViep3) derived from these association domains of IgG1 efficiently inhibited binding of FCN1-N with IgG1. Therefore, these peptides could have beneficial clinical applications. Because the association domains fluctuated depending on the dissection sites of the IgG1 molecule (Figs 5 and 6), it will be necessary to perform X-ray crystallographic analysis in a future study to determine the exact domains responsible for the association between FCN1 and IgG1. After three-dimensional structures are determined, it will become possible to design small molecules that can potently inhibit the FCN1–IgG1 association. Such molecules, which would be more stable in serum than the iViep peptides, could be used as drugs to treat vasculitis in the clinic.

IVIG non-responders are prone to develop coronary artery lesions^18^, a major risk factor for development of aneurysms; at least 20% of IVIG treated KD patients are IVIG-refractory. Because early diagnosis of IVIG-refractory patients would enable timely management of other anti-inflammatory therapies, previous studies have sought to identify markers capable of distinguishing IVIG responders from non-responders^19^; at present, however, no such markers are available. When we compared the gene-expression profiles of thirteen responders and six non-responders, we found no genes specifically up- or down-regulated in non-responders (Fig. 1, S2). Wb of PBMCs revealed that four (KD1, KD12, KD14, and KD16) of six non-responders had elevated FCN1 levels, whereas two others (KD2 and KD11) had a dramatically elevated FCN1 level at d7; however, some of the responders (KD4, KD21, KD24, and KD25) exhibited similar patterns (Fig. 3). Examination of serum FCN1 levels revealed incomplete depletion of FCN1 in all five non-responders (KD32, KD33, KD34, KD36, and KD37) even after IVIG treatment, although some responders (KD38, KD40, KD41, and KD43) also had similar residual FCN1 levels (Fig. 4). Thus, FCN1 levels in PBMCs and serum may serve as diagnostic markers to distinguish non-responders. Although we found three novel single nucleotide variants (SNVs) in the KD patients, none of them distinguished responders from non-responders (Fig. S13).

In addition to KD, IVIG is also useful for the treatment of several rheumatic conditions, including dermatomyositis and specific subgroups of rheumatoid arthritis patients who are refractory to anti-cytokine blockers or rituximab, a monoclonal antibody against the CD20 protein, which is expressed primarily detected on the surface of B cells^10, 20^. Notably, combination therapy with IVIG and rituximab is effective in treating patients with chronic active antibody-mediated rejection, and is recommended for delaying the progression of symptoms without serious complications^21^. Combination therapy with infliximab, a monoclonal antibody against TNF-alpha that suppresses cytokine-mediated inflammation, is therapeutically effective in IVIG-refractory KD patients^2^. These observations suggest that a monoclonal antibody against an unknown target protein of IVIG might serve as a therapeutic molecule in IVIG-refractory KD patients. Given our identification of FCN1 as a molecular target of IVIG in KD patients, we propose that a monoclonal anti-FCN1 antibody could be useful in combination therapy. We plan to investigate this possibility in future research.

## Patients, Materials and Methods

### Human subjects and ethical considerations

Twenty-eight KD patients were enrolled at Toho University Omori Medical Center; the genders and ages of the patients are provided in Figure S1. The study was reviewed and approved by the Research Ethics Committee of Toho University (A16050_27053), and written informed consent was obtained from all participants. We confirm that all experiments were performed in accordance with relevant guidelines and regulations. Diagnosis of KD was established according to standard criteria proposed by the Japan KD Research Committee^22^.

### DNA microarray analysis

We used the PAXgene Blood RNA Kit (Qiagen) for purification of RNA from PBMCs of KD patients and HVs. Quality of RNA samples was determined using an RNA 6000 Nano LabChip Kit (p/n 5065-4476) on an Agilent 2100 Bioanalyzer (G2940BA; Agilent Technologies). Total RNA (500 ng) isolated from PBMCs of each KD patient was examined along with pooled RNA from HVs. Each patient’s RNA and the pooled HV RNA were reverse-transcribed using oligo-dT primers containing the T7 RNA polymerase promoter sequence, and the resultant cDNA was subjected to *in vitro* transcription using T7 RNA polymerase; fluorescently tagged precursors were added to label each sample with Cy3 or Cy5 (CyDye, Amersham Pharmacia Biotech). Cy5-labeled cRNA from post-IVIG KD patients (1625 ng) was mixed with the same amount of Cy3-labeled cRNA from pre-IVIG KD patients. Hybridization, rinsing, scanning, and gene analysis were conducted using Agilent Whole Human Genome Microarrays (4 × 44 K G4112F). The Cy5/Cy3 ratio was calculated for each probe, and the results were analyzed using the Subio Basic Plug-in (v1.6; Subio Inc., Aichi, Japan), which allows visualization of microarray data in the form of heat maps and line graphs. The detailed microarray data have been deposited in the Gene Expression Omnibus (GEO; www.ncbi.nlm.nih.gov/geo) database (accession number GSE73577).

### Preparation of protein extracts of PBMCs and Wb analysis

Cell lysates were prepared from PBMCs using LeukoCatch^®23^ (BioAcademia, Osaka, http://www.bioacademia.co.jp/en/product_list.php?srch_keyword=leukocatch), which is convenient because it allows removal of hemoglobin from blood samples within a few minutes. Briefly, about 5 ml of blood was applied to LeukoCatch^®^ to attach PBMC protein extracts to the LeukoCatch^®^ filter, which was washed several times with ice-cold phosphate-buffered saline. The extract was eluted in lysis buffe^23^. After lysates were subjected to SDS-PAGE, the resolved proteins were transferred to PVDF membranes, which were blocked and immunoblotted with the indicated antibodies in Tris-buffered saline and Tween 20 containing 5% nonfat milk. Immunoreactive protein bands were visualized using Western Lightning Plus ECL Pro reagents (PerkinElmer).

### Antibodies and reagents

Antibodies against the following proteins were purchased from the indicated companies: OMG, METTL7B, MS4A7, and haptoglobin β (Santa Cruz Biotechnology); HSP90 and PRMT5 (Cell Signaling); S100A8 and haptoglobin α (Sigma-Aldrich); FAP (Abnova); α-Tubulin (MBL); GAPDH (Fitzgerald); C3 (BioLegend); C1q (Novus); and C4 (AssayPro).

## Electronic supplementary material

Supplementary information
