# Supplementary material for: FCN1 (M-ficolin), which directly associates with immunoglobulin G1, is a molecular target of intravenous immunoglobulin therapy for Kawasaki disease
Source: Sci Rep. 2017 Sep 12;7:11334. doi: 10.1038/s41598-017-11108-0 (PMC5595863; doi:10.1038/s41598-017-11108-0)
Supplement: Supplementary file 1 — Supplementary information [file 41598_2017_11108_MOESM1_ESM.pdf]

# FCN1 (M-ficolin), which directly associates with immunoglobulin G1, is a molecular target of intravenous immunoglobulin therapy for Kawasaki disease

**Daisuke Okuzaki, Kaori Ota, Shin-ichi Takatsuki, Yukari Akiyoshi, Kazuyuki Naoi, Norikazu Yabuta Tsutomu Saji and Hiroshi Nojima**

## Supplementary Information

### Supplementary Materials and Methods

**Expression profiling using quantitative reverse transcription-polymerase chain reaction (qRT-PCR).** We performed qRT-PCR on an ABI PRISM 7900 (PE Applied Biosystems, Foster City, CA) using the Assay-on-Demand TaqMan probe and gene-specific primers. For *HP*, *METTL7B*, *HPR*, *LOC100506229*, *FCN1,* and *FAP*, assay kits (ID Hs00605928_g1, Hs00378551_m1, Hs00750565_s1, Hs01591421_m1, Hs00157572_m1, and Hs00990806_m1, respectively) were purchased from PE Applied Biosystems. The following oligonucleotides were used as primers and probes for *GAPDH*: *GAPDH* forward primer, 5'-CCATCAATGACCCCTTCATTG-3'; *GAPDH* reverse primer, 5'-TCTCGCTCCTGGAAGATGGT-3'; and *GAPDH* TaqMan probe, 5'-VIC-ACCTCAACTACATGGTTTAC-MGBNFQ-3'. Total RNA (500 ng) was reverse-transcribed using the High Capacity cDNA Archive Kit (ABI). The resultant cDNA was used as a template for PCR in a 20 μL reaction containing 10 μL of 2× Master Mix (TaKaRa, Otsu, Japan). PCR conditions were as follows: initial denaturation at 95°C for 10 min, followed by 40 cycles of denaturation at 95°C for 15 s and annealing/extension at 60°C for 1 min. Each sample was assayed in quadruplicate, and the median threshold cycle ($C_T$) values were used to calculate fold changes between the treated and control samples. A standard curve was generated from the amplification data for each primer using serial dilutions of PBMC RNA as the template. Fold change values were normalized to the corresponding levels of *GAPDH* levels using the standard curve method.

**Plasmids.** Double-stranded DNA of full human FCN1 cDNA was chemically synthesized by GenScript USA Inc. Human IgG1 cDNAs were purchased from New England Biolabs Inc. and Origene Inc. Plasmids encoding FCN1-N, FCN1-C, and dissected fragments of IgG1 were obtained by PCR-based amplification of these cDNAs and cloned into the *Asc*I and *Not*I sites of mammalian expression vectors (pCMV6myc and p3FLAG) and a bacterial expression vector (pGST6P). All amplified sequences were confirmed by DNA sequencing.

**IP/Wb to examine the association between Flag-FCN1 and Myc-IgG1 proteins.** Plasmid DNAs designed to express Flag-FCN1 or Myc-IgG1 proteins under the control of the cytomegalovirus (CMV) promoter were transfected to 293T cells using Lipofectamine (Thermo Fisher Scientific). After 48 h incubation in DMEM, each cell extract (dissolved in 500 µL of TNE250 including protease inhibitors: TNEI) was mixed with anti-Myc antibody, and then subjected to inversion mixing process on a rotator overnight at 4°C to facilitate complete binding of FCN-N and IgG1 proteins. After brief centrifugation (4°C; 4,900 × *g*; 2 min), the precipitate was rinsed again three times with 500 µL TNEI. To the final precipitate, 15 µL of 2× SDS-PAGE sample buffer was added; the sample was boiled in hot water for 7 min, and then subjected to Wb using anti-Myc antibody (IP/Wb) or anti-FLAG antibody (loading control).

**Pull-down experiments using GST-FCN1-N.** Plasmid DNA designed to express GST-FCN1-N fragment was cloned into the *Asc*I and *Not*I sites of GST-fused protein expression vector (pGST6P)[23] derived from pGEX6P (Amersham Pharmacia), and then transfected into *E. coli* (BL21 RIL) using competent cells prepared according to the SEM protocol[24]. Single colonies that expressed GST-FCN1-N proteins with the highest efficiency were selected. Next, glutathione–Sepharose beads (15 µL) used for affinity purification were mixed with cell extracts from 293T cells (10 µg) expressing Myc-IgG1 fragments and then subjected to inversion mixing on a rotator overnight at 4°C to facilitate complete binding of GST-FCN-N and IgG1 proteins. After brief centrifugation (4°C; 4,900 × *g*; 2 min), the precipitate was rinsed again three times with 500 µL TNEI. To the final precipitate, 15 µL 2× SDS-PAGE sample buffer was added; the sample was boiled in hot water for 7 min, and then subjected to Wb using anti-Myc antibody (IP/Wb) or anti-FLAG antibody (loading control).

**Inhibition of FCN-N and IgG1 fragments by synthetic peptides.** Glutathione–Sepharose beads (15 µL) containing affinity purified GST-FCN1-N proteins were rinsed twice with TNE, and the precipitate was dissolved in 500 µL TNEI. Next, chemically synthesized peptides were added, and the samples were subjected to inversion mixing on a rotator for 1 h at 4°C. To this mixture was added 10 µg cell extract from 293T cells expressing Myc-IgG1 fragments, and the sample was again subjected to inversion mixing for 1 h at 4°C to allow competitive binding of peptides and Myc-IgG1-3d or Myc-IgG1-CH1p fragments. After brief centrifugation (4°C; 4,900 × *g*; 2 min), the precipitate was rinsed again three times with 500 µL TNEI. To the final precipitate, 15 µL of 2× SDS-PAGE sample buffer was added; the sample was boiled in hot water for 7 min, and then subjected to Wb using anti-Myc antibody (IP/Wb) or anti-FLAG antibody (loading control).

**SNVs in the *FCN1* gene of KD patients.** DNA sequence of *FCN1* cDNA was determined using cDNA fragments amplified by PCR using mRNA of KD patients as substrates.

**Statistical analysis.** Error bars for all data represent standard deviation (SD) from the mean. P-values were calculated using Student's t-test.

## Supplementary References


23. Suzuki, H., Yabuta, N., Okada, N., Torigata, K., Aylon, Y., Oren, M. & Nojima, H. Lats2 phosphorylates p21/CDKN1A after UV irradiation and regulates apoptosis. *J Cell Sci.* **126**, 4358-4368 (2013). doi: 10.1242/jcs.125815. PubMed PMID:23886938.
24. Inoue, H., Nojima, H. & Okayama, H. High efficiency transformation of *Escherichia coli* with plasmids. *Gene* **96**, 23-28 (1990). PMID: 2265755.

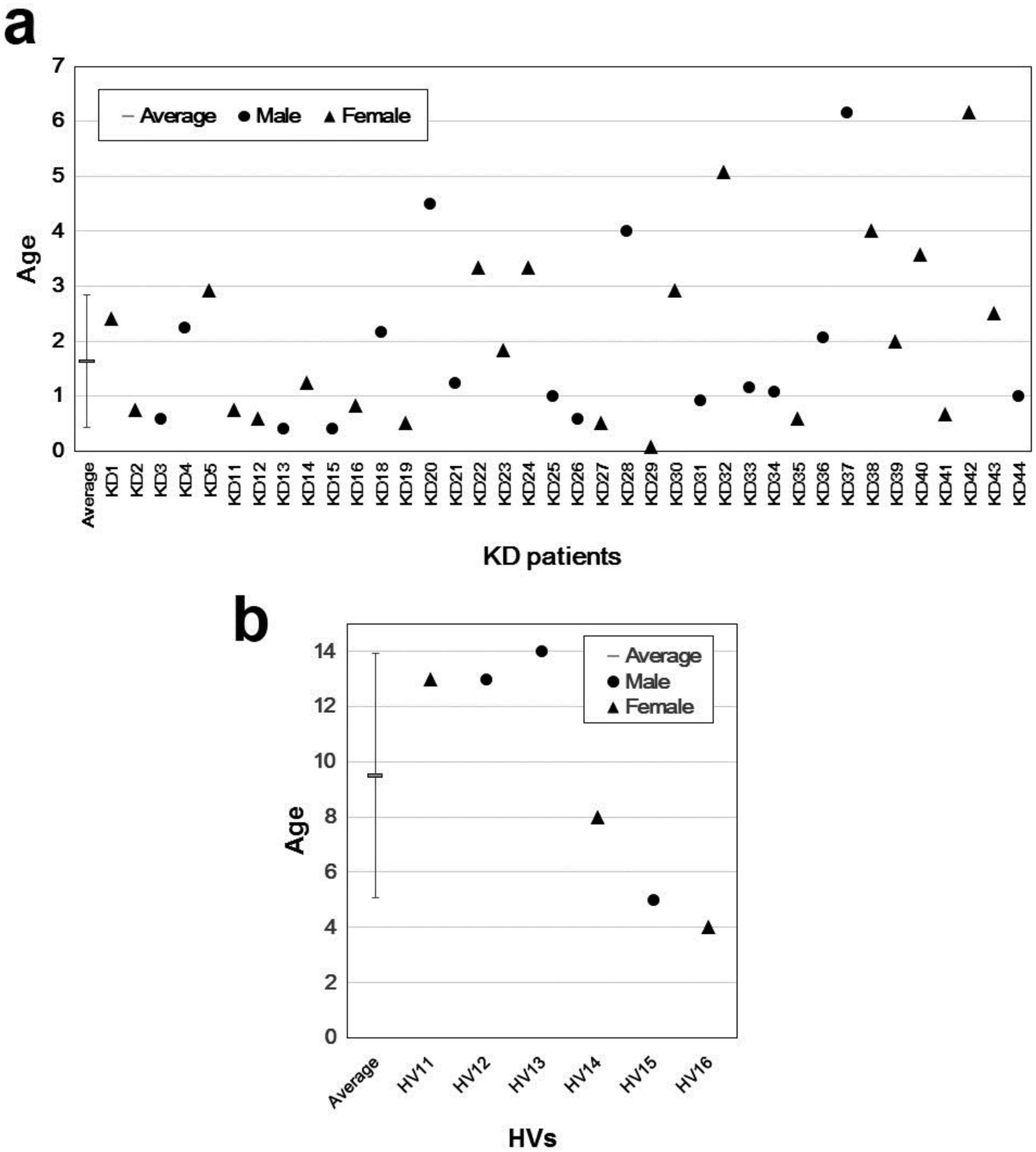


**Figure S1. Gender and age distributions of BD patients (a) and HVs (b).** Females and males are indicated by circles and triangles, respectively.

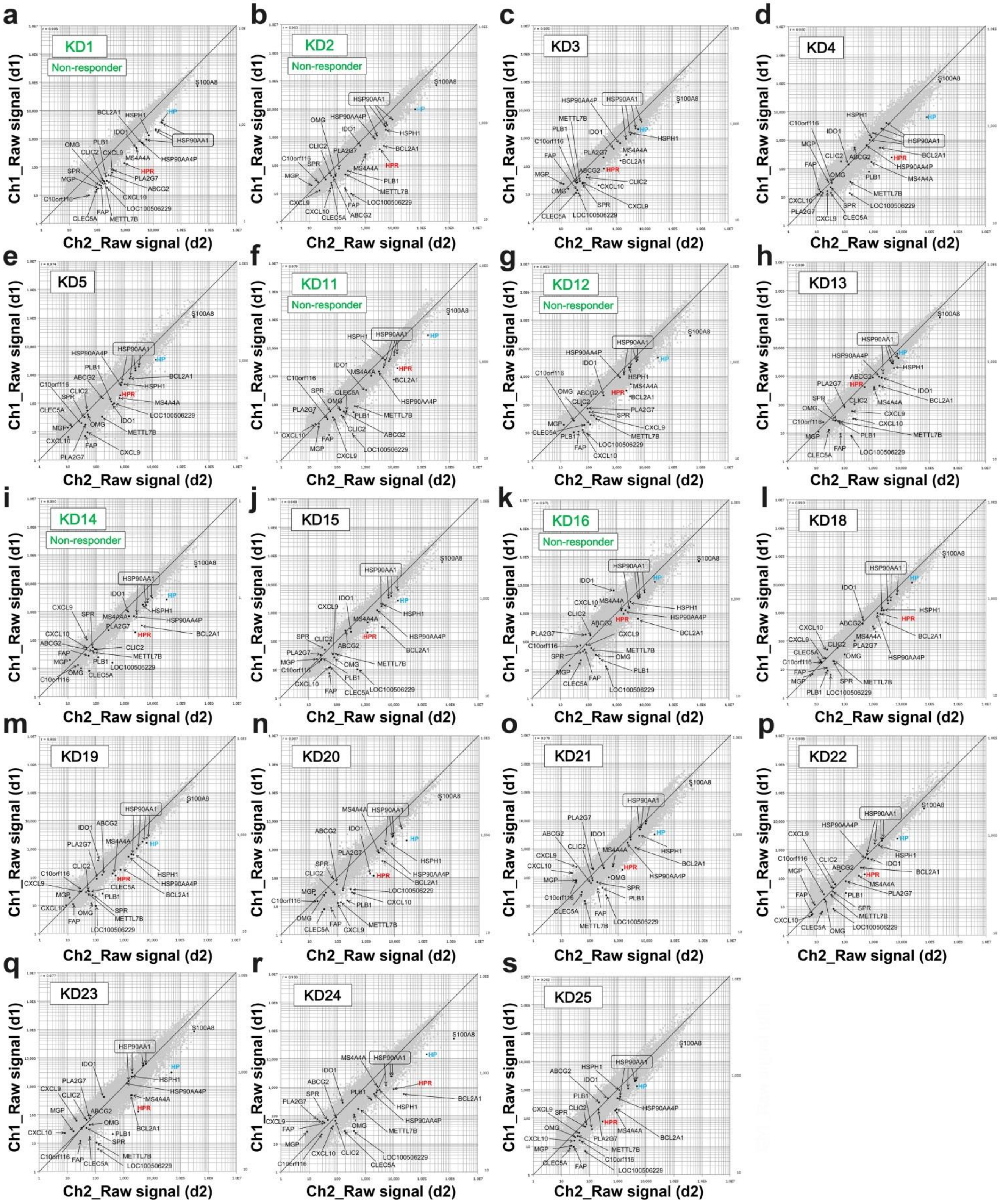


**Figure S2. Scatter plots of genes highlighted in Fig. 1 for individual KD patients. (a–s)** Scatter plot for each KD patient. The y-axis shows the log value of hybridization signal intensity obtained from the microarray data for each KD patient. The y-axis and x-axis show log2[hybridization signal intensity] of microarray data from d1 and d2, respectively, for the indicated KD patient. Females and males are indicated by circles and triangles, respectively.

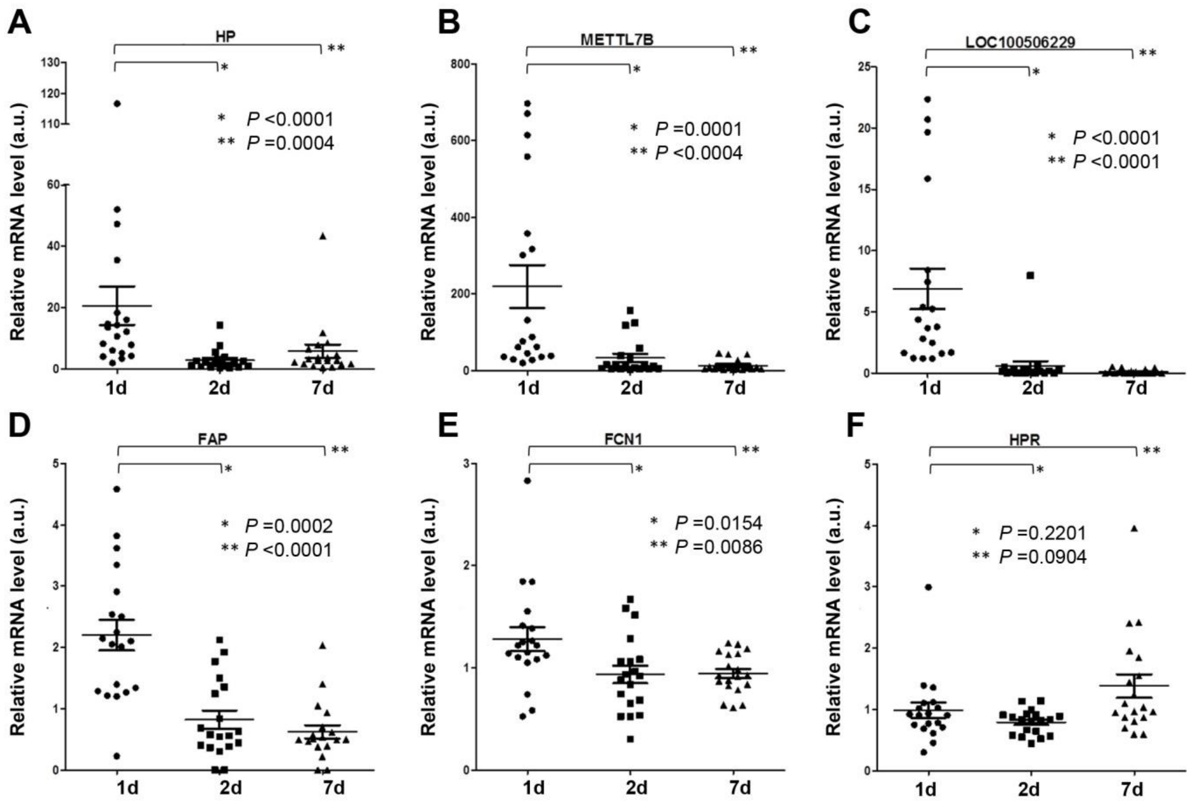


**Figure S3. Dot plot of raw qRT-PCR raw data.** Relative mRNA levels of *HP* **(a)**, *METTL7B* **(b)**, *LOC100506229* **(c)**, *FAP* **(d)**, *FCN1* **(e)**, *and HPR* **(f)** were determined by qRT-PCR using using purified RNA from each KD patient on the indicated day. Day 1 (d1) indicates blood collected before IVIG treatment; d2 or d7 indicates blood collected 2–3 days or 6–8 days after IVIG treatment, respectively. Horizontal bar in each dot plot denotes average value of 19 KD patients, with standard deviation bars. The vertical axis indicates mRNA level (arbitrary units, a.u.) relative to that at 1d, which was fixed at 1.0 a.u. Average and standard deviation values (error bars) are shown.

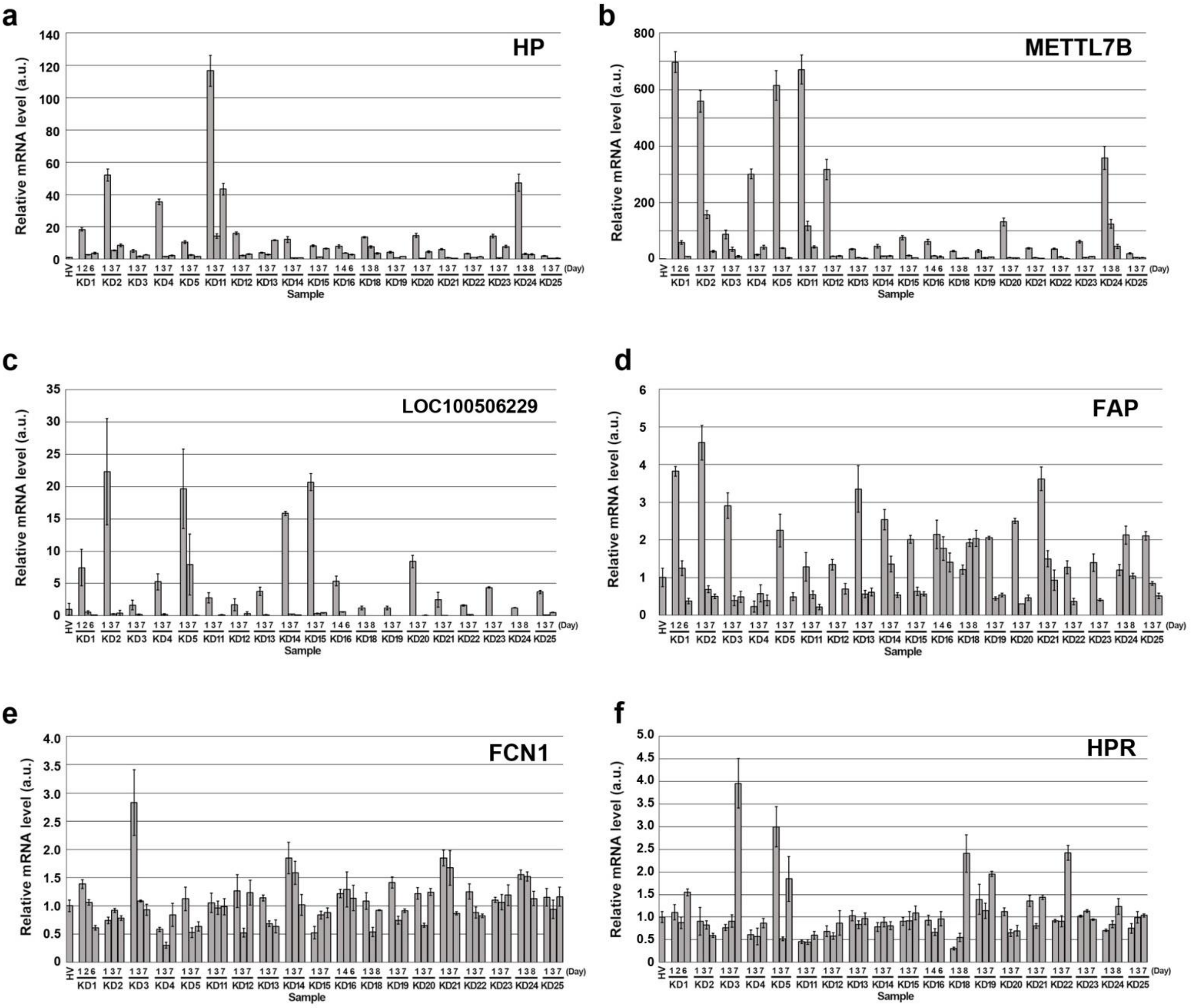


**Figure S4. Bar graph of raw qRT-PCR raw data.** Relative mRNA levels of *HP* **(a)**, *METTL7B* **(b)**, *LOC100506229* **(c)**, *FAP* **(d)**, *FCN1* **(e)**, *and HPR* **(f)** were determined by qRT-PCR using purified RNA from HVs (leftmost column) and from each KD patient (lower x-axis labels) on the indicated day. Number for each bar (Day) indicates the date when blood was collected from each patient. The vertical axis indicates mRNA level (a.u.) relative to that of HV, which was fixed at 1.0 a.u.

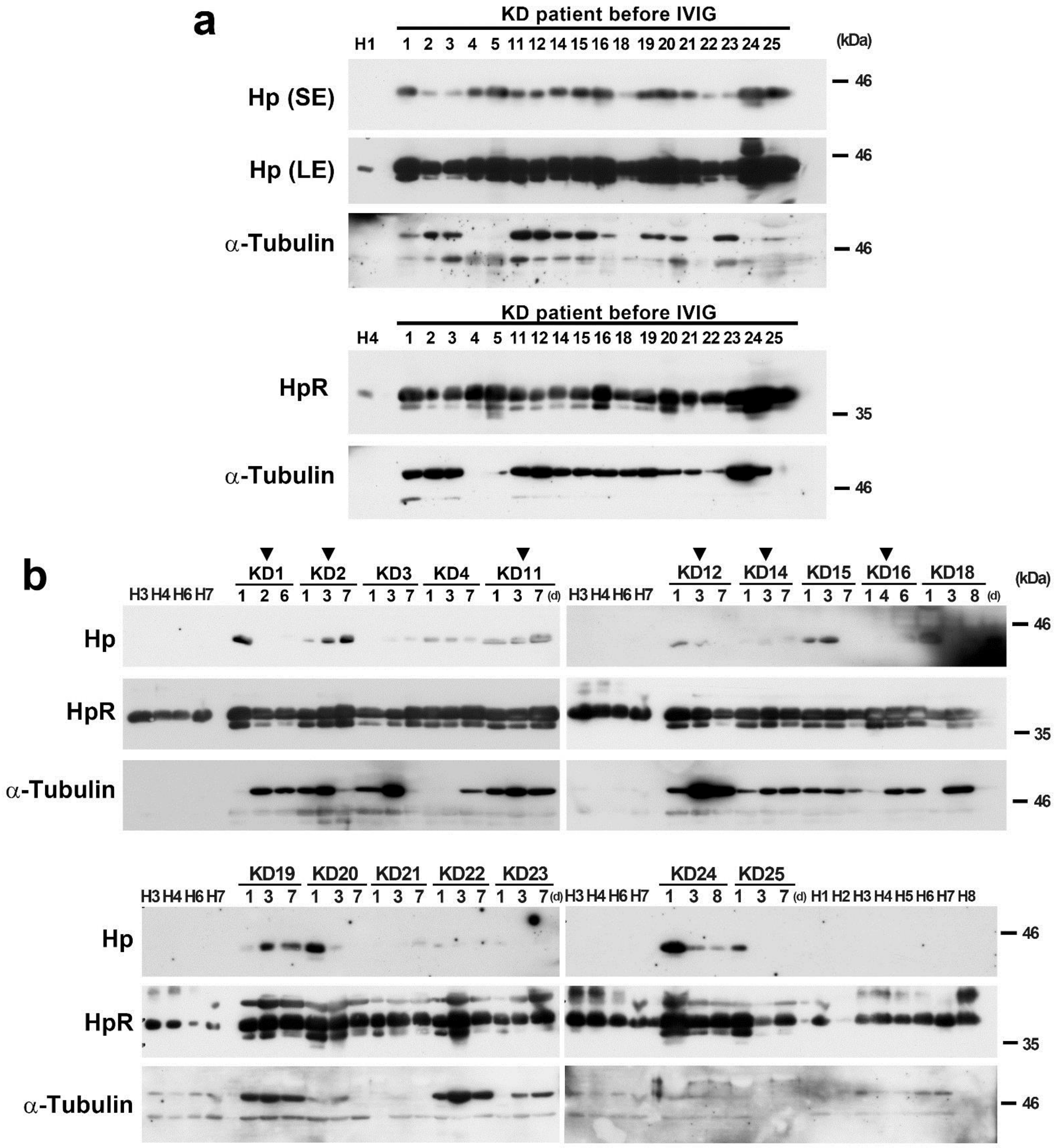


**Figure S5. Western blot analysis to detect expression of Hp and HpR proteins in PBMCs of KD patients.** For Hp, images obtained by short exposure (SE) or long exposure (LE) are displayed. Protein levels of PBMCs from healthy volunteers (H1, H3–H7) were used to highlight altered expression levels in KD patients. α-tubulin was used as a loading control. Number for each bar (d) indicates the date when blood was collected from each patient.

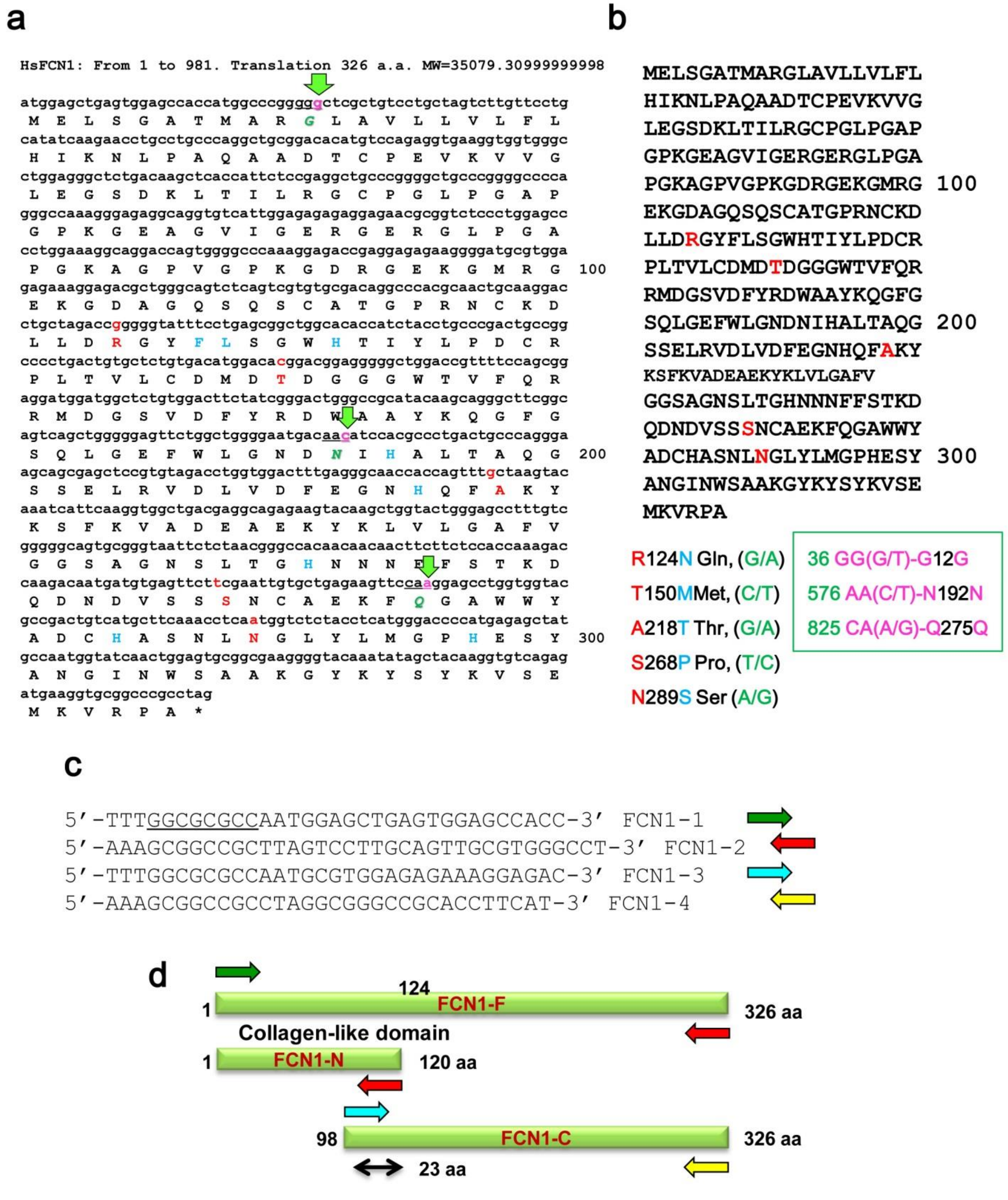


**Figure S6. Structure of human FCN1.** (a) Nucleotide and amino acid (aa) sequences of human *FCN1* mRNA and FCN1 protein. Nucleotides and amino acids shown in red font denote the sites of known single-nucleotide variants (SNVs) of human *FCN1*. Vertical green arrows indicate the sites of novel SNVs that we detected in our KD patients (see Fig. S13). (b) Amino acid sequence and sites of SNVs of human FCN1. (c) Nucleotide sequences of primers used for PCR-based construction of plasmids that express FCN1-F, FCN1-N, and FCN1-C proteins. (d) Schematic representations of FCN1-F, FCN1-N, and FCN1-C proteins. Arrows indicate the sites and directions of primers used for PCR.

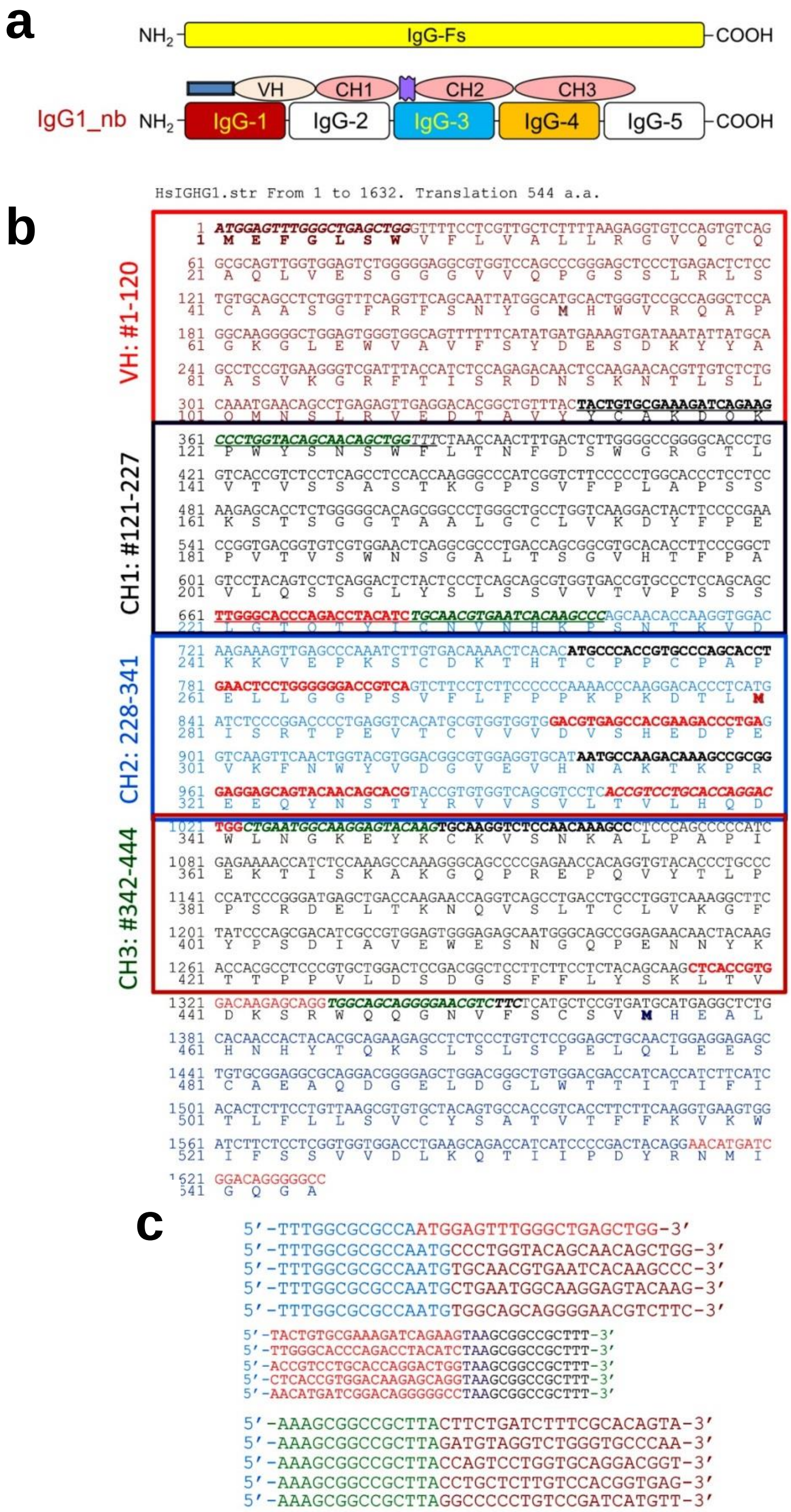


**Figure S7. Structure of human IgG1_nb.** (a) Schematic presentation of human IgG1 cDNA (yellow box) obtained from NEB, which harbors an additional C-terminal sequence due to an artificial mutation at the original termination codon. The relative locations of VH, CH1, CH2, and CH3 domains plus a hinge region (purple box) and the dissected domains (IgG-1, IgG-2, IgG-3, IgG-4, and IgG-5) are shown. (b) Nucleotide sequences of human IgG1 cDNA and its translated protein; the VH, CH1, CH2, and CH3 domains are encircled by colored boxes. Nucleotide sequences of primers used for PCR-based construction of plasmids expressing dissected IgG1 proteins are indicated in colored and bold font. (c) Nucleotide sequences of primers used for PCR-based construction of plasmids expressing the dissected domains of IgG1 (IgG-1, IgG-2, IgG-3, IgG-4, and IgG-5).

**a**

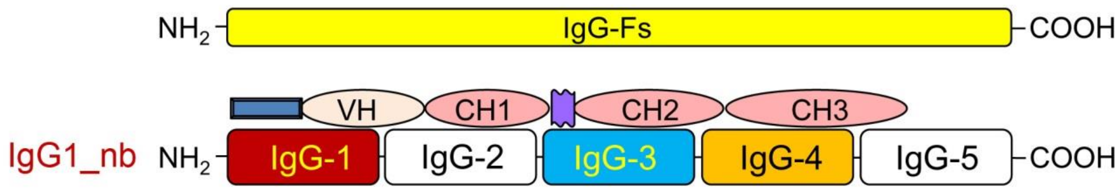


**b**

**IgG1-1:**

```
              *         *         *         *         *         *         *         *         *         *
   1>~~~~~~~~~~~~~~~~~~~~~~~~~~~~~~~~~~~~~~~~~~~~~~~~~~~~~~~~~~~~~~~~~~~~~~~~~~~~~~~~ATGGAGTTTGGGCTGAGCTG>20
 301>TGGGCGACCTCACCATGGAGCAAAAGCTCATTTCTGAAGAGGACTTGAATTCCTGCAGCCCGGGGGATCCGGCGCGCCAATGGAGTTTGGGCTGAGCTG>400
              *         *         *         *         *         *         *         *         *         *
  21>GGTTTTCCTCGTTGCTCTTTTAAGAGGTGTCCAGTGTCAGGCGCAGTTGGTGGAGTCTGGGGGAGGCGTGGTCCAGCCCGGGAGCTCCCTGAGACTCTCC>120
 401>GGTTTTCCTCGTTGCTCTTTTAAGAGGTGTCCAGTGTCAGGCGCAGTTGGTGGAGTCTGGGGGAGGCGTGGTCCAGCCCGGGAGCTCCCTGAGACTCTCC>500
              *         *         *         *         *         *         *         *         *         *
 121>TGTGCAGCCTCTGGTTTCAGGTTCAGCAATTATGGCATGCACTGGGTCCGCCAGGCTCCAGGCAAGGGGCTGGAGTGGGTGGCAGTTTTTTCATATGATG>220
 501>TGTGCAGCCTCTGGTTTCAGGTTCAGCAATTATGGCATGCACTGGGTCCGCCAGGCTCCAGGCAAGGGGCTGGAGTGGGTGGCAGTTTTTTCATATGATG>600
              *         *         *         *         *         *         *         *         *         *
 221>AAAGTGATAAATATTATGCAGCCTCCGTGAAGGGTCGATTTACCATCTCCAGAGACAACTCCAAGAACACGTTGTCTCTGCAAATGAACAGCCTGAGAGT>320
 601>AAAGTGATAAATATTATGCAGCCTCCGTGAAGGGTCGATTTACCATCTCCAGAGACAACTCCAAGAACACGTTGTCTCTGCAAATGAACAGCCTGAGAGT>700
              *         *         *         *         *         *         *         *         *         *
 321>TGAGGACACGGCTGTTTACTACTGTGCGAAAGATCAGAAGCCCTGGTACAGCAACAGCTGGTTTCTAACCAACTTTGACTCTTGGGGCCGGGGCACCCTG>420
 701>TGAGGACACGGCTGTTTACTACTGTGCGAAAGATCAGAAG------------------------------------------------------------>740
```

**IgG1-2:**

```
              *         *         *         *         *         *         *         *         *         *
299>TGCAAATGAACAGCCTGAGAGTTGAGGACACGGCTGTTTACTACTGTGCGAAAGATCAGAAGCCCTGGTACAGCAACAGCTGGTTTCTAACCAACTTTGA>398
 34>-------------------------------------------------------------GCCCTGGTACAGCAACAGCTGGTTTCTAACCAACTTTGA>72
      *         *         *         *         *         *         *         *         *         *
399>CTCTTGGGGCCGGGGCACCCTGGTCACCGTCTCCTCAGCCTCCACCAAGGGCCCATCGGTCTTCCCCCTGGCACCCTCCTCCAAGAGCACCTCTGGGGGC>498
 73>CTCTTGGGGCCGGGGCACCCTGGTCACCGTCTCCTCAGCCTCCACCAAGGGCCCATCGGTCTTCCCCCTGGCACCCTCCTCCAAGAGCACCTCTGGGGGC>172
      *         *         *         *         *         *         *         *         *         *
499>ACAGCGGCCCTGGGCTGCCTGGTCAAGGACTACTTCCCCGAACCGGTGACGGTGTCGTGGAACTCAGGCGCCCTGACCAGCGGCGTGCACACCTTCCCGG>598
173>ACAGCGGCCCTGGGCTGCCTGGTCAAGGACTACTTCCCCGAACCGGTGACGGTGTCGTGGAACTCAGGCGCCCTGACCAGCGGCGTGCACACCTTCCCGG>272
      *         *         *         *         *         *         *         *         *         *
599>CTGTCCTACAGTCCTCAGGACTCTACTCCCTCAGCAGCGTGGTGACCGTGCCCTCCAGCAGCTTGGGCACCCAGACCTACATCTGCAACGTGAATCACAA>698
273>CTGTCCTACAGTCCTCAGGACTCTACTCCCTCAGCAGCGTGGTGACCGTGCCCTCCAGCAGCTTGGGCACCCAGACCTACATCT---------------->3
```

**IgG1-3:**

```
601>GTCCTACAGTCCTCAGGACTCTACTCCCTCAGCAGCGTGGTGACCGTGCCCTCCAGCAGCTTGGGCACCCAGACCTACATCTGCAACGTGAATCACAAGC>700
  4>---------------------------------------------------------------------------------TGCAACGTGAATCACAAGC>22
             *         *         *         *         *         *         *         *         *         *
701>CCAGCAACACCAAGGTGGACAAGAAAGTTGAGCCCAAATCTTGTGACAAAACTCACACATGCCCACCGTGCCCAGCACCTGAACTCCTGGGGGGACCGTC>800
 23>CCAGCAACACCAAGGTGGACAAGAAAGTTGAGCCCAAATCTTGTGACAAAACTCACACATGCCCACCGTGCCCAGCACCTGAACTCCTGGGGGGACCGTC>122
             *         *         *         *         *         *         *         *         *         *
801>AGTCTTCCTCTTCCCCCCAAAACCCAAGGACACCCTCATGATCTCCCGGACCCCTGAGGTCACATGCGTGGTGGTGGACGTGAGCCACGAAGACCCTGAG>900
123>AGTCTTCCTCTTCCCCCCAAAACCCAAGGACACCCTCATGATCTCCCGGACCCCTGAGGTCACATGCGTGGTGGTGGACGTGAGCCACGAAGACCCTGAG>222
             *         *         *         *         *         *         *         *         *         *
901>GTCAAGTTCAACTGGTACGTGGACGGCGTGGAGGTGCATAATGCCAAGACAAAGCCGCGGGAGGAGCAGTACAACAGCACGTACCGTGTGGTCAGCGTCC>1000
223>GTCAAGTTCAACTGGTACGTGGACGGCGTGGAGGTGCATAATGCCAAGACAAAGCCGCGGGAGGAGCAGTACAACAGCACGTACCGTGTGGTCAGCGTCC>322
             *         *         *         *         *         *         *         *         *         *
1001>TCACCGTCCTGCACCAGGACTGGCTGAATGGCAAGGAGTACAAGTGCAAGGTCTCCAACAAAGCCCTCCCAGCCCCCATCGAGAAAACCATCTCCAAAGC>1100
 323>TCACCGTCCT-CACCAGGACTGG~~~~~~~~~~~~~~~~~~~~~~~~~~~~~~~~~~~~~~~~~~~~~~~~~~~~~~~~~~~~~~~~~~~~~~~~~~~~~~>344
```

**IgG1-4:**

```
1001>TCACCGTCCTGCACCAGGACTGGCTGAATGGCAAGGAGTACAAGTGCAAGGTCTCCAACAAAGCCCTCCCAGCCCCCATCGAGAAAACCATCTCCAAAGC>1100
   1>~~~~~~~~~~~~~~~~~~~~~~~CTGAATGGCAAGGAGTACAAGTGCAAGGTCTCCAACAAAGCCCTCCCAGCCCCCATCGAGAAAACCATCTCCAAAGC>77
              *         *         *         *         *         *         *         *         *         *
1101>CAAAGGGCAGCCCCGAGAACCACAGGTGTACACCCTGCCCCCATCCCGGGATGAGCTGACCAAGAACCAGGTCAGCCTGACCTGCCTGGTCAAAGGCTTC>1200
  78>CAAAGGGCAGCCCCGAGAACCACAGGTGTACACCCTGCCCCCATCCCGGGATGAGCTGACCAAGAACCAGGTCAGCCTGACCTGCCTGGTCAAAGGCTTC>177
              *         *         *         *         *         *         *         *         *         *
1201>TATCCCAGCGACATCGCCGTGGAGTGGGAGAGCAATGGGCAGCCGGAGAACAACTACAAGACCACGCCTCCCGTGCTGGACTCCGACGGCTCCTTCTTCC>1300
 178>TATCCCAGCGACATCGCCGTGGAGTGGGAGAGCAATGGGCAGCCGGAGAACAACTACAAGACCACGCCTCCCGTGCTGGACTCCGACGGCTCCTTCTTCC>277
              *         *         *         *         *         *         *         *         *         *
1301>TCTACAGCAAGCTCACCGTGGACAAGAGCAGGTGGCAGCAGGGGAACGTCTTCTCATGCTCCGTGATGCATGAGGCTCTGCACAACCACTACACGCAGAA>1400
 278>TCTACAGCAAGCTCACCGTGGACAAGAGCAGG~~~~~~~~~~~~~~~~~~~~~~~~~~~~~~~~~~~~~~~~~~~~~~~~~~~~~~~~~~~~~~~~~~~~>309
```

**IgG1-5:**

```
1301>TCTACAGCAAGCTCACCGTGGACAAGAGCAGGTGGCAGCAGGGGAACGTCTTCTCATGCTCCGTGATGCATGAGGCTCTGCACAACCACTACACGCAGAA>1400
   1>~~~~~~~~~~~~~~~~~~~~~~~~~~~~~~~~TGGCAGCAGGGGAACGTCTTCTCATGCTCCGTGATGCATGAGGCTCTGCACAACCACTACACGCAGAA>68
              *         *         *         *         *         *         *         *         *         *
1401>GAGCCTCTCCCTGTCTCCGGAGCTGCAACTGGAGGAGAGCTGTGCGGAGGCGCAGGACGGGGAGCTGGACGGGCTGTGGACGACCATCACCATCTTCATC>1500
  69>GAGCCTCTCCCTGTCTCCGGAGCTGCAACTGGAGGAGAGCTGTGCGGAGGCGCAGGACGGGGAGCTGGACGGGCTGTGGACGACCATCACCATCTTCATC>168
              *         *         *         *         *         *         *         *         *         *
1501>ACACTCTTCCTGTTAAGCGTGTGCTACAGTGCCACCGTCACCTTCTTCAAGGTGAAGTGGATCTTCTCCTCGGTGGTGGACCTGAAGCAGACCATCATCC>1600
 169>ACACTCTTCCTGTTAAGCGTGTGCTACAGTGCCACCGTCACCTTCTTCAAGGTGAAGTGGATCTTCTCCTCGGTGGTGGACCTGAAGCAGACCATCATCC>268
              *         *         *
1601>CCGACTACAGGAACATGATCGGACAGGGGGCCTAA>1635
 269>CCGACTACAGGAACATGATCGGACAGGGGGCCTAA>303
```

**Figure S8. Confirmation of nucleotide sequences of the dissected domains of IgG1_nb.** (a) Schematic presentation of human IgG1 cDNA (yellow box) obtained from NEB relative to the dissected domains of IgG1. (b) Confirmation of nucleotide sequences of theB and locations of the VH, CH1, CH2, and CH3 domains plus J chain (purple box) re cDNA inserts sandwiched between 5' primers (red font) and 3' primers (green font; reverse sequence) used for PCR-based construction of plasmids expressing the dissected domains of IgG1 (IgG-1, IgG-2, IgG-3, IgG-4, and IgG-5). These plasmid DNAs were used in FCN1-N binding assays.

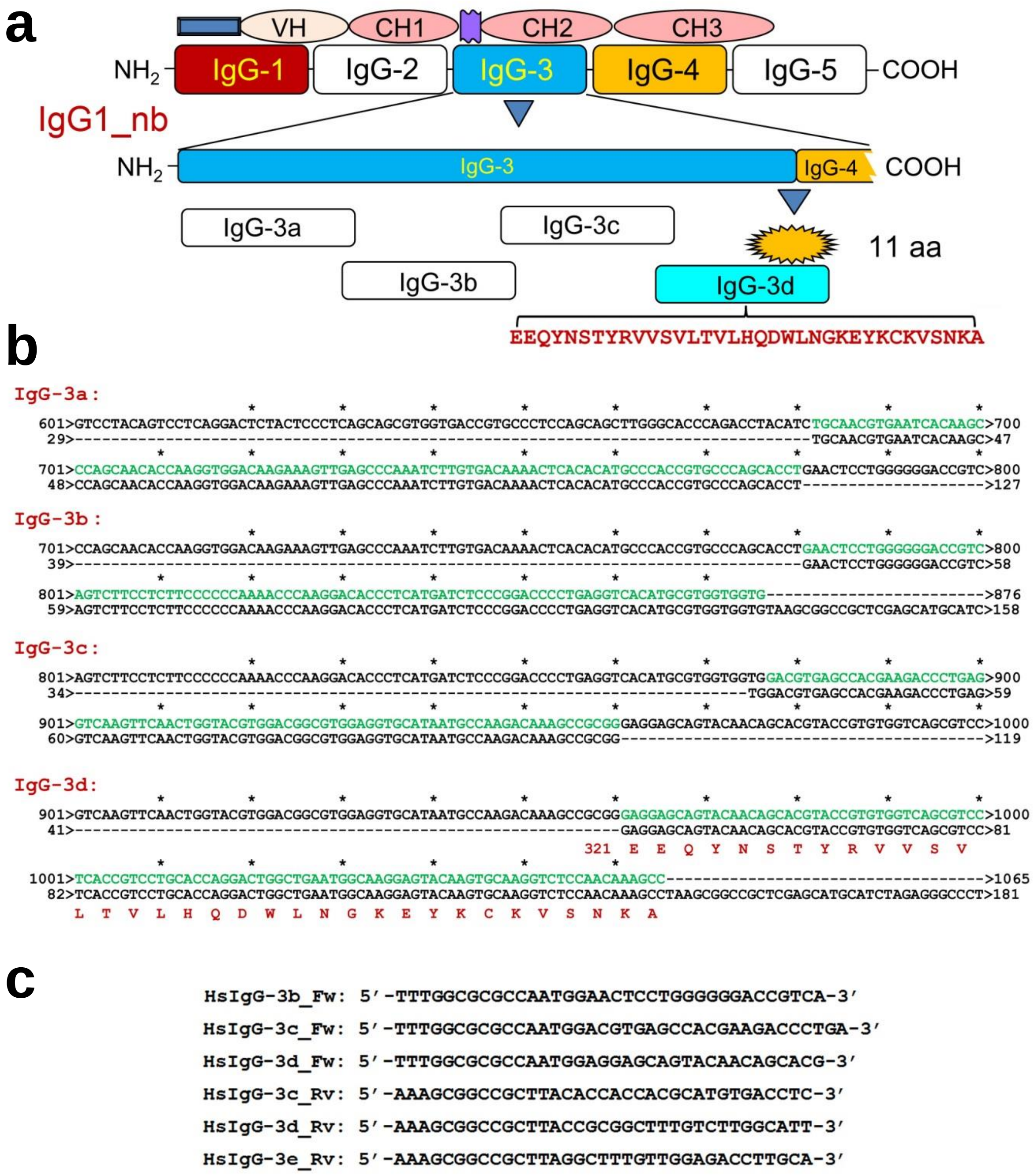


**Figure S9. Confirmation of nucleotide sequences of the dissected domains of IgG-3.** (a) Schematic presentation of human IgG1 cDNA (yellow box) obtained from NEB and locations of VH, CH1, CH2, and CH3 domains plus J chain (purple box) relative to the IgG1 dissected domains. (b) Confirmation of nucleotide sequences of the cDNA inserts sandwiched between 5’ primers (red font) and 3’ primers (green font; reverse sequence) used for PCR-based construction of plasmids expressing the dissected domains of IgG1 (IgG-1, IgG-2, IgG-3, IgG-4, and IgG-5). These plasmid DNAs were used in FCN1 binding assays. (c) Nucleotide sequences of primers used for PCR-based construction of plasmids. Fw: forward primer. Rv: reverse primer.

a

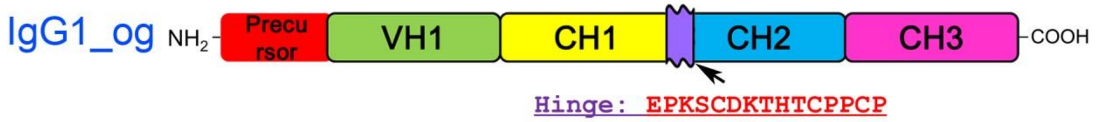


b

```
1    agctctgagagaggagccttagccctggattccaaggcctatccacttggtgatcagcactgagcaccgaggattcaccatggaactggggctccgctgggttttccttgttgctctt
1                                                                                         M E L G L R W V F L V A L

121  ttagaaggtgtccactgtgaggtgcagttggtggagtctgggggaggcctggtcaagccggggggtccctgagactctcctgtgcagcctctggattcagcttcagttattataccatg
41   L E G V H C E V Q L V E S G G G L V K P G G S L R L S C A A S G F S F S Y Y T M

241  aactgggtccgccaggctccagggaaggggctggagtgggtctcatccatcactattagtagtacttacctatcttacgcagactcagtgaagggccggttcaccatctccagagacaac
81   N W V R Q A P G K G L E W V S S I T I S S T Y L S Y A D S V K G R F T I S R D N

361  gccaagaatgtgctgtatctgcacatgaacagcctgagagtcgaggacacggctgtctattattgtacgagggatagggtagtggctggttcggatccaaactttgactattggggcccg
121  A K N V L Y L H M N S L R V E D T A V Y Y C T R D R V V A G S D P N F D Y W G P

481  ggaaccctagtcaccgtctcctcagcctccaccaagggcccatcggtcttccccctggcaccctcctccaagagcacctctgggggcacagcggccctgggctgcctggtcaaggactac
161  G T L V T V S S A S T K G P S V F P L A P S S K S T S G G T A A L G C L V K D Y

601  ttccccgaaccggtgacggtgtcgtggaactcaggcgccctgaccagcggcgtgcacaccttcccggctgtcctacagtcctcaggactctactccctcagcagcgtggtgaccgtgccc
201  F P E P V T V S W N S G A L T S G V H T F P A V L Q S S G L Y S L S S V V T V P

721  tccagcagcttgggcacccagacctacatctgcaacgtgaatcacaagcccagcaacaccaaggtggacaagaaagtt gagcccaaatcttgtgacaaaactcacacatgcccaccgtgc
241  S S S L G T Q T Y I C N V N H K P S N T K V D K K V E P K S C D K T H T C P P C

841  ccagcacctgaactcctggggggaccgtcagtcttcctcttccccccaaaacccaaggacaccctcatgatctcccggacccctgaggtcacatgcgtggtggtggacgtgagccacgaa
281  P A P E L L G G P S V F L F P P K P K D T L M I S R T P E V T C V V V D V S H E

961  gaccctgaggtcaagttcaactggtacgtggacggcgtggaggtgcataatgccaagacaaagccgcgggaggagcagtacaacagcacgtaccgtgtggtcagcgtcctcaccgtcctg
321  D P E V K F N W Y V D G V E V H N A K T K P R E E Q Y N S T Y R V V S V L T V L

1081 caccaggactggctgaatggcaaggagtacaagtgcaaggtctccaacaaagccctcccagcccccatcgagaaaaccatctccaaagccaaagggcagccccgagaaccacaggtgtac
361  H Q D W L N G K E Y K C K V S N K A L P A P I E K T I S K A K G Q P R E P Q V Y

1201 accctgcccccatcccgggatgagctgaccaagaaccaggtcagcctgacctgcctggtcaaaggcttctatcccagcgacatcgccgtggagtgggagagcaatgggcagccggagaac
401  T L P P S R D E L T K N Q V S L T C L V K G F Y P S D I A V E W E S N G Q P E N

1321 aactacaagaccacgcctcccgtgctggactccgacggctccttcttcctctacagcaagctcaccgtggacaagagcaggtggcagcaggggaacgtcttctcatgctccgtgatgcat
441  N Y K T T P P V L D S D G S F F L Y S K L T V D K S R W Q Q G N V F S C S V M H

1441 gaggctctgcacaaccactacacgcagaagagcctctccctgtctccgggtaaatgagtgcgacggccggcaagcccccgctccccgggctctcgcggtcgcacgaggatgcttggcacg
481  E A L H N H Y T Q K S L S L S P G K

1561 taccccgtgtacatacttcccgggcgcccagcatggaaataaagcacccagcgctgccctgggcccctg
```

c

| | |
|---|---|
| HsIgG1#2-PrFw | 5'-TTTGGCGCGCCAATGGAACTGGGGCTCCGCTGG-3' |
| HsIgG1#2-VhFw | 5'-TTTGGCGCGCCAATGGTGCAGTTGGTGGAGTCTGGG-3' |
| HsIgG1#2-CH1Fw | 5'-TTTGGCGCGCCAATGGCCTCCACCAAGGGCCCATCG-3' |
| HsIgG1#2-CH2Fw | 5'-TTTGGCGCGCCAATGGCACCTGAACTCCTGGGGGGA-3' |
| HsIgG1#2-CH3Fw | 5'-TTTGGCGCGCCAATGGGGCAGCCCCGAGAACCACAG-3' |
| HsIgG1#2-PrRv | 5'-AAAGCGGCCGCTTACTCACAGTGGACACCTTCTAA-3' |
| HsIgG1#2-VhRv | 5'-AAAGCGGCCGCTTATGAGGAGACGGTGACTAGGGT-3' |
| HsIgG1#2-CH1Rv | 5'-AAAGCGGCCGCTTATGGGCACGGTGGGCATGTGTG-3' |
| HsIgG1#2-CH2Rv | 5'-AAAGCGGCCGCTTATTTGGCTTTGGAGATGGTTTT-3' |
| HsIgG1#2-CH3Rv | 5'-AAAGCGGCCGCTTATTTACCCGGAGACAGGGAGAG-3' |

**Figure S10. Structure of human IgG1_og.** (a) Schematic representation of human IgG1 cDNA, obtained from OriGene. Locations of precursor, VH, CH1, CH2, and CH3 domains plus hinge region (purple box) are shown. (b) Nucleotide sequences of human IgG1 cDNA and its translated protein. Amino acids of the region of VH1 homologous to the other VH1 regions are shown in turquoise and crimson font. (c) Nucleotide sequences of primers used for PCR-based construction of plasmids (see Fig. S11). Fw: forward primer. Rv: reverse primer.

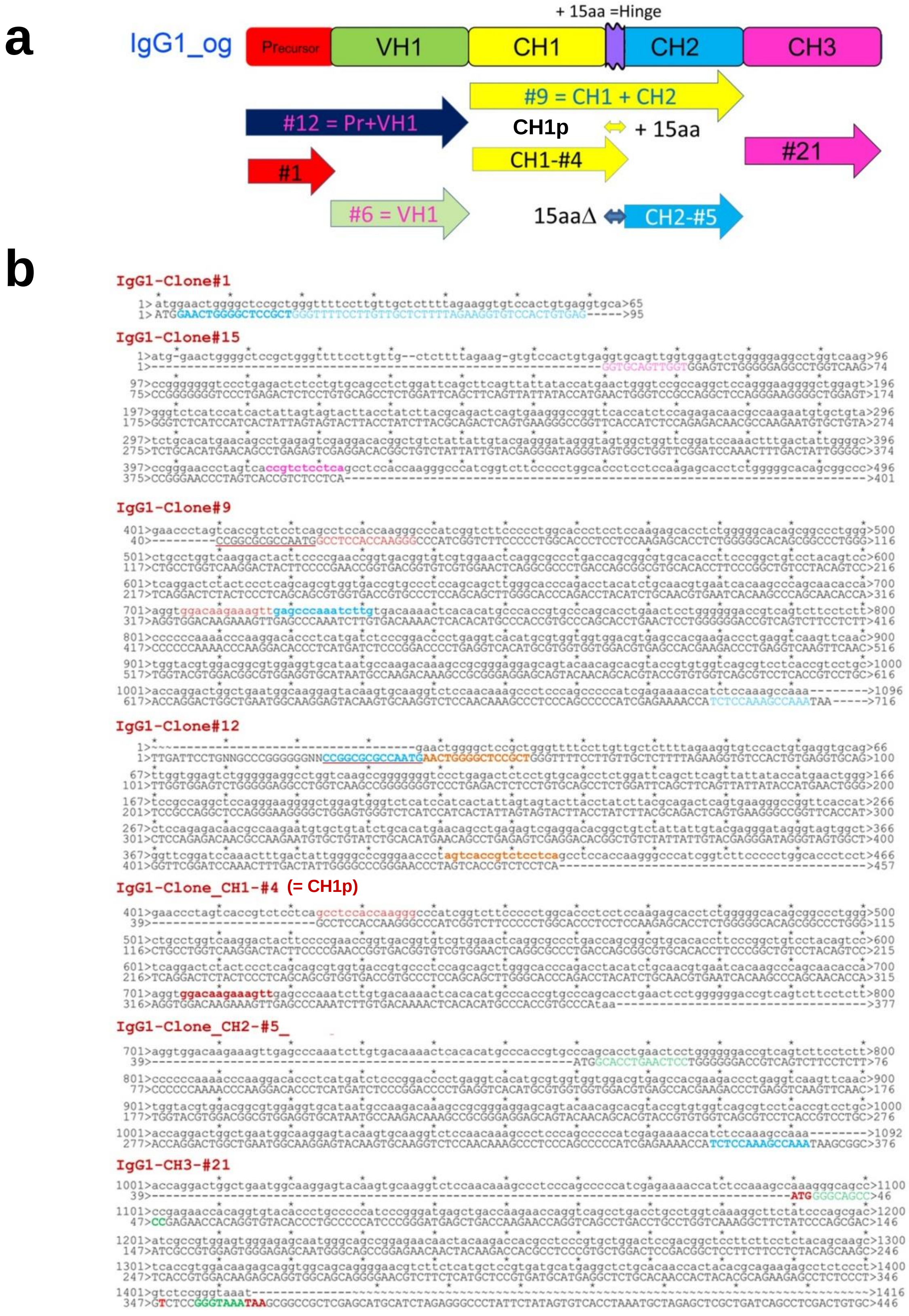


**Figure S11. Confirmation of nucleotide sequences in the dissected domains of IgG1_og.** (a) Schematic representation of human IgG1 cDNA obtained from OriGene and locations of dissected domains. (b) Confirmation of nucleotide sequences of the cDNA inserts sandwiched between 5' primers (red font) and 3' primers (green font; reverse sequence) used for PCR-based construction of plasmids. These plasmid DNAs were used in FCN1 binding assays. Nucleotide sequences used for primers are shown in colored font. Underlined (red) nucleotides sequences indicate the *Asc*I restriction site used to insert the cDNA into the vector and the translation initiation codon (ATG).

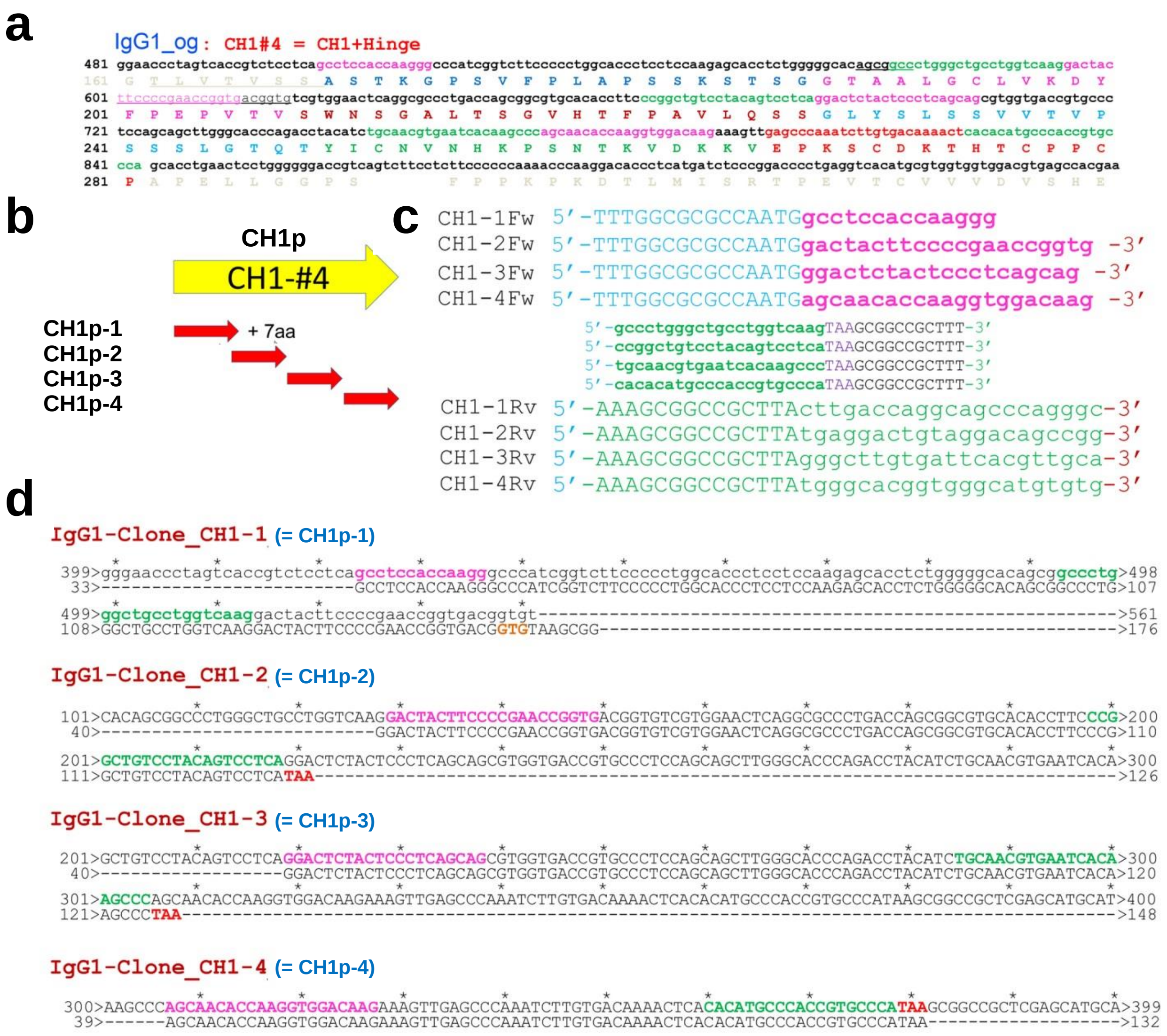


**Figure S12. Confirmation of nucleotide sequences in the dissected domains of CH1p of IgG1_og.** (a) Nucleotide and amino acid sequences of CH1p (CH1-#4) clone. Each dissected region is highlighted in a colored font. (b) Schematic representation of CH1p (CH1-#4) and four dissected domains (CH1-1, -2, -3, and -4). (c) Nucleotide sequences of primers used for PCR-based construction of plasmids. Fw: forward primer. Rv: reverse primer. (d) Confirmation of nucleotide sequences of the cDNA inserts sandwiched between 5’ primers (red font) and 3’ primers (green font; reverse sequence) used for PCR-based construction of plasmids. These plasmid DNAs were used in FCN-N binding assays.

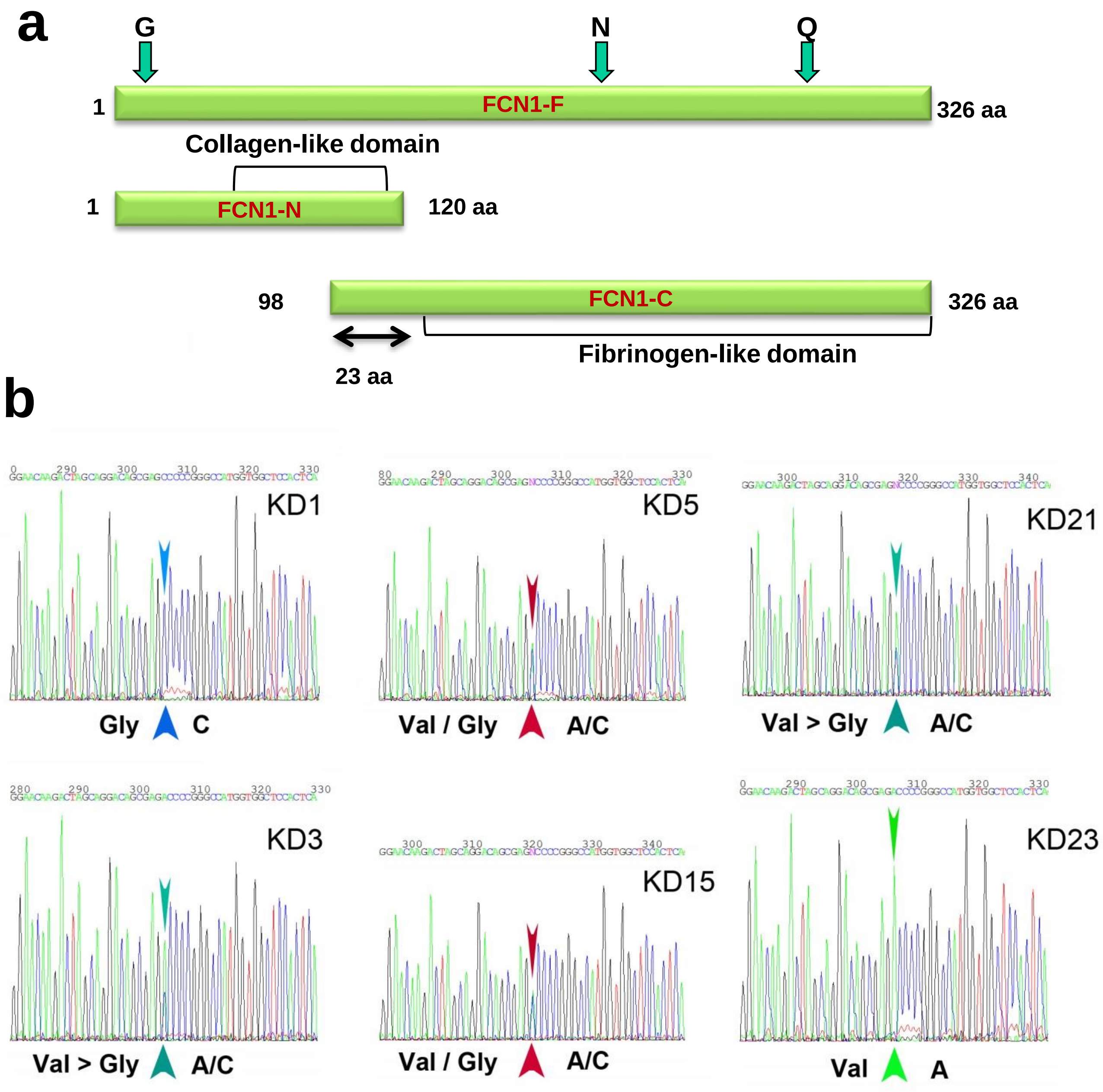


**Figure S13. Three novel SNVs detected in the *FCN1* gene of KD patients.** (a) Schematic presentation of FCN1-F (full size), FCN1-N (collagen-like domain), and FCN1-C (fibrinogen-like domain). KD patients harbored three novel SNVs (G, N, and Q) at the indicated sites (green arrows). (b) Typical nucleotide sequencing ladders around the SNVs of KD patients.
